# multi-GPA-Tree: Statistical approach for pleiotropy informed and functional annotation tree guided prioritization of GWAS results

**DOI:** 10.1371/journal.pcbi.1011686

**Published:** 2023-12-07

**Authors:** Aastha Khatiwada, Ayse Selen Yilmaz, Bethany J. Wolf, Maciej Pietrzak, Dongjun Chung

**Affiliations:** 1 Department of Biostatistics and Bioinformatics, National Jewish Health, Denver, Colorado, United States of America; 2 Department of Biomedical Informatics, The Ohio State University, Columbus, Ohio, United States of America; 3 Department of Public Health Sciences, Medical University of South Carolina, Charleston, South Carolina, United States of America; 4 Pelotonia Institute for Immuno-Oncology, The James Comprehensive Cancer Center, The Ohio State University, Columbus, Ohio, United States of America; University of Pennsylvania, UNITED STATES

## Abstract

Genome-wide association studies (GWAS) have successfully identified over two hundred thousand genotype-trait associations. Yet some challenges remain. First, complex traits are often associated with many single nucleotide polymorphisms (SNPs), most with small or moderate effect sizes, making them difficult to detect. Second, many complex traits share a common genetic basis due to ‘pleiotropy’ and and though few methods consider it, leveraging pleiotropy can improve statistical power to detect genotype-trait associations with weaker effect sizes. Third, currently available statistical methods are limited in explaining the functional mechanisms through which genetic variants are associated with specific or multiple traits. We propose multi-GPA-Tree to address these challenges. The multi-GPA-Tree approach can identify risk SNPs associated with single as well as multiple traits while also identifying the combinations of functional annotations that can explain the mechanisms through which risk-associated SNPs are linked with the traits. First, we implemented simulation studies to evaluate the proposed multi-GPA-Tree method and compared its performance with existing statistical approaches. The results indicate that multi-GPA-Tree outperforms existing statistical approaches in detecting risk-associated SNPs for multiple traits. Second, we applied multi-GPA-Tree to a systemic lupus erythematosus (SLE) and rheumatoid arthritis (RA), and to a Crohn’s disease (CD) and ulcertive colitis (UC) GWAS, and functional annotation data including GenoSkyline and GenoSkylinePlus. Our results demonstrate that multi-GPA-Tree can be a powerful tool that improves association mapping while facilitating understanding of the underlying genetic architecture of complex traits and potential mechanisms linking risk-associated SNPs with complex traits.

## Author summary

In spite of continued success in developing statistical methodologies that integrate GWAS summary statistics and functional annotation data, existing methods are unable to pinpoint the interactions between functional annotations that influence one or more traits. Hence, the underlying interactions between biological mechanisms linking risk-associated SNPs to traits remain unknown. We propose multi-GPA-Tree to identify risk-associated SNPs and the combinations of functional annotations related to one or more trait risk-associated SNPs. Notably, multi-GPA-Tree requires only GWAS *p*-value summary statistics, instead of individual level genotype-phenotype data, making it more viable to implement. Compared to the existing state-of-the-art methods, multi-GPA-Tree showed improved performance in simulation studies and validated results for several auto-immune diseases in real data application. These combined results suggest that multi-GPA-Tree is an effective tool for integrative analysis and can potentially be valuable to clinical genomic researchers for hypothesis generation and validation.

## Introduction

Increasing interest in identifying genomic regions associated with complex traits has resulted in a substantial increase in the number of reported GWAS studies and genotype-trait associations (https://www.ebi.ac.uk/gwas/) [1]. Identification of previously unknown genotype-trait associations has improved estimation of heritability (genetic variation within a trait) for many complex traits. However, two major challenges persist. First, some proportion of heritability remains missing for many traits due to unidentified genotype-trait associations [2–4]. Polygenicity, a phenomenon that causes genetic variants to be associated with traits with weak or moderate effect sizes [5, 6] is a plausible explanation for missing heritibility. The impact of polygenicity can theoretically be reduced by recruiting a larger GWAS sample size to increase statistical power to detect weak and moderate associations; however, large sample recruitment often requires more resources and is not always feasible due to limited trait prevalence in the population [7]. An alternative to increasing sample size to improve statistical power is to exploit the pleiotropic relationship (shared genetic basis) between two or more traits by simultaneously integrating GWAS association summary statistics for multiple traits [8–10]. GWAS summary statistics are readily available to use as input through public data repositories [1, 11] and are good proxy to using individual-level genotype-phenotype data that are harder to obtain. Second, majority of the GWAS identified loci are located in the non-coding regions [12], making it difficult to understand the functional mechanisms related to identified genotype-trait associations. For example, in autoimmune diseases, about 90% of the causal genetic variants lie in non-coding regions, a bulk of which are located in regulatory DNA regions [13, 14]. As such, utilizing genomic functional annotation information that can provide information related to different types of histone modifications, epigenetic and cell- and tissue-specific changes, etc. can be useful to decode the functional mechanisms linking risk-associated genetic variants to traits [15–17]. Therefore, integrative analysis of genetic data with genomic functional annotation data is a promising direction.

Statistical methods built on the foundation of data integration approaches not only utilize information that are readily available in public data repositories but also overcome the challenges posed by polygenicity while simultaneously providing insights about underlying functional mechanisms related to one or more traits. Therefore, they are more advantageous and efficient. In recognizing the potential to enhance statistical power to detect associations through data integration approaches, several statistical methods focused on GWAS summary statistics have been developed [8, 10, 15, 16–19]. These methods can broadly be classified into three distinct categories.

The first category of methods exploit the pleiotropic relationship between two or more distinct traits by simultaneously integrating multiple GWAS association *p*-values together. Two favored methods in this category are the pleiotropy-informed conditional FDR approach [8] and the graph-GPA approach [10]. The unifying goal of the two methods is to improve statistical power to prioritize one or more trait risk-associated SNPs. The conditional FDR approach shows improved detection of risk-associated SNPs for two psychiatric disorders, schizophrenia and bipolar disorder. Despite easy implementation of this approach, the lack of a model-based framework in estimating conditional FDR compromises the power to detect non-null associations and also to infer the properties of the non-null distribution. Moreover, this approach can only integrate a small number of GWAS traits. In contrast, graph-GPA can integrate large number of GWAS traits using a hidden Markov random field framework and its usefulness is demonstrated by integrating 12 traits (five psychiatric disorders, three autoimmune traits, two lipid-related traits and two cardiovascular traits) where clinically related traits form closely connected clusters. However, both methods fail to inform about functional relevance of risk-associated SNPs due to their inability to integrate functional annotations in their application.

The second category of methods integrate individual GWAS data with genotype-related functional annotation data. Two cutting-edge approaches in this category include the latent sparse mixed model (LSMM) approach [16] and the covariate modulated false discovery rate (cmFDR) approach [17]. In LSMM, functional annotations are integrated using a logistic mixed effects model framework where genic- and cell-type specific functional annotations are assumed to respectively have fixed and random effects and a sparse structure is imposed on the random effects to adaptively select cell-type specific functional annotations that may be relevant to a trait etiology. The application of LSMM discovered substantial enrichment of blood-related cell-type specific annotations for autoimmune diseases like systemic lupus erythematosus, rheumatoid arthritis, ulcerative colitis and Crohn’s disease. Similar to LSMM, the cmFDR approach is a parametric method that integrates GWAS summary statistics and functional annotation information where functional annotation information provide ‘prior information’ in a parametric two-group mixture model. The cmFDR approach assumes that compared to SNPs that are not functionally relevant, SNPs that are functionally relevant have a lower false discovery rate, and are associated with the trait. However, both cmFDR and LSMM do not exploit the pleiotropic relationship between traits with similar etiology to improve power to detect associations.

Finally, the third category of statistical methods combine the first two category criteria and integrate multiple GWAS trait data together with genotype-related functional annotation data. Two well known methods in this category include the genetic analysis incorporating pleiotropy and annotation (GPA) approach [18] and the more recent latent probit model (LPM) approach [19]. GPA employs a unified statistical framework to integrate genetically correlated GWAS traits by leveraging pleiotropy and functional annotation data to perform joint analysis of multiple traits. Similar to GPA, the three main goals of LPM are to identify the pleiotropic relationship between multiple traits by estimating the correlation between the traits, to identify the effect of functional annotations, and to improve the power to identify risk-associated SNPs for one or more traits. In both methods, the number of parameters that are included in the model increases significantly as the number of GWAS traits and functional annotations increase, rendering their implementation statistically and computationally challenging. Moreover, although methods in the second and third category can perform enrichment analysis on individual annotations, these methods do not consider interactions between the annotations, and therefore are limited in informing about the combined functional pathways through which genetic variants are associated with one or more traits. While some of these methods can theoretically be extended to include interactions between functional annotations to evaluate the combined functional effect of annotations, they retain the burden of knowing a priori the interactions that are of interest. Therefore, a method that can perform variable selection to identify relevant functional annotations or combinations of functional annotations from a large group of annotations that are linked to genetic variants associated with one or more traits is vitally important.

To address the statistical challenges and limitations described above, our team recently published a novel statistical approach called GPA-Tree [20] that simultaneously performs association mapping and identification of interactions between functional annotations. However, GPA-Tree does not exploit the pleiotropic relationship between two or more traits to improve association mapping power. In this work, we address the limitations of the GPA-Tree approach by proposing a new approach called multi-GPA-Tree. The multi-GPA-Tree approach is a novel statistical method based on a hierarchical modeling architecture, integrated with a multivariate regression tree algorithm [21]. It exploits the pleiotropic relationship between traits with similar etiology to prioritize one or more trait-associated SNPs while simultaneously identifying key combinations of functional annotations related to the mechanisms through which one or more trait-associated SNPs influence the trait/s. Our comprehensive simulation studies and real data applications show that multi-GPA-Tree consistently improves statistical power to detect one or more trait-associated SNPs and also effectively identifies biologically important combinations of functional annotations. The multi-GPA-Tree approach takes GWAS summary statistics for multiple traits and functional annotation information for the GWAS genetic variants as input, and can be implemented using the R package ‘multiGPATree’.

## Materials and methods

### Overview of the multi-GPA-Tree approach

Let **Y**_*M* × *D*_ be a matrix of genotype-trait association *p*-values for *i* = 1, 2, ⋯, *M* SNPs and *d* = 1, 2, ⋯, *D* traits where *Y*_*id*_ denotes the *p*-value for the association of the *i*^*th*^ SNP with the *d*^*th*^ trait.

$$\begin{array}{c} Y=\left(Y_{.1},\ldots ,Y_{.D}\right)={\left(\begin{array}{ccc} y_{11} & \cdots & y_{1D}\\ \vdots & \ddots & \vdots \\ y_{M1} & \cdots & y_{MD} \end{array}\right)}_{M\times D} \end{array}$$

We also assume K binary annotations (**A**) for each SNP.

$$\begin{array}{c} A=\left(A_{.1},\ldots ,A_{.K}\right)={\left(\begin{array}{ccc} a_{11} & \cdots & a_{1K}\\ \vdots & \ddots & \vdots \\ a_{M1} & \cdots & a_{MK} \end{array}\right)}_{M\times K}\;\text{,}\;\;\;\text{where} \end{array}$$

$$\begin{array}{c} a_{ik}=\{\begin{array}{cc} 0, & \text{if}\;i^{th}\;\text{SNP}\;\text{is}\;\text{not}\;\text{annotated}\;\text{in}\;\text{the}\;k^{th}\;\text{annotation}\\ 1, & \text{if}\;i^{th}\;\text{SNP}\;\text{is}\;\text{annotated}\;\text{in}\;\text{the}\;k^{th}\;\text{annotation} \end{array} \end{array}$$

To improve the power to identify risk-associated SNPs for one or more traits, GWAS association *p*-values for *D* traits (**Y**) are integrated with functional annotations data (**A**). The impact of functional annotations in modeling the relationship between GWAS traits and SNPs is characterized by defining a matrix **Z**_*M*×2^*D*^_ ∈ {0, 1} of latent binary variables where **Z**_*i*_ is a vector of length 2^*D*^ and indicates whether the *i*^*th*^ SNP is null or non-null for the *D* traits. Here, we present the model for the case of two GWAS traits (*D* = 2) to simplify notations.

Let $Y\in R^{M\times 2}$ be the matrix of GWAS association *p*-values for two traits where *Y*_*i*1_ and *Y*_*i*2_ are the *p*-values for the association between the *i*^*th*^ SNP and traits 1 and 2, respectively. The latent binary vector is defined as **Z**_*i*_ = {*Z*_*i*00_, *Z*_*i*10_, *Z*_*i*01_, *Z*_*i*11_} for the *i*^*th*^ SNP, where *Z*_*i*00_ = 1 indicates the *i*^*th*^ SNP is null for both traits, *Z*_*i*10_ = 1 indicates the *i*^*th*^ SNP is non-null for trait 1 and null for trait 2, *Z*_*i*01_ = 1 indicates the *i*^*th*^ SNP is null for trait 1 and non-null for trait 2 and *Z*_*i*11_ = 1 indicates the *i*^*th*^ SNP is non-null for both traits. We assume that a SNP can only be in one of the four states such that $\sum_{l\in \{00,10,01,11\}}Z_{il}=1$. The densities for SNPs in the null and non-null groups for both traits are assumed to come from *U*[0, 1] and *Beta*(*α*_*d*_, 1) distributions, where 0 < *α*_*d*_ < 1 and *d* = 1, 2, as proposed in [18]. The distributions are defined as shown below.

$$\begin{array}{c} \begin{array}{cc} & Y_{i1}\left|Z_{i00}=1∼U\left[0,1\right]\;Y_{i2}\right|Z_{i00}=0∼U\left[0,1\right]\\ & Y_{i1}\left|Z_{i10}=1∼Beta\left(\alpha_{1},1\right)\;Y_{i2}\right|Z_{i10}=1∼U\left[0,1\right]\\ & Y_{i1}\left|Z_{i01}=1∼U\left[0,1\right]\;Y_{i2}\right|Z_{i01}=1∼Beta\left(\alpha_{2},1\right)\\ & Y_{i1}\left|Z_{i11}=1∼Beta\left(\alpha_{1},1\right)\;Y_{i2}\right|Z_{i11}=1∼Beta\left(\alpha_{2},1\right), \end{array} \end{array}$$

where 0 < *α*_1_, *α*_2_ < 1. Finally, the functional annotation data **A** is integrated with the GWAS summary statistics data **Y** by defining a function *f* that is a combination of functional annotations **A** and relating it to the multivariate expectation of latent **Z** as given in Eq 1.

$$\begin{array}{c} \begin{array}{c} P\left(Z_{il}=1;a_{i1},\ldots ,a_{iK}\right)=\text{f}\left(a_{i1},\ldots ,a_{iK}\right),\text{where}\;l\in \left\{00,10,01,11\right\} \end{array} \end{array}$$ (1)

For notational convenience we let ***θ*** = (*α*_1_, *α*_2_) and denote *P*(*Z*_*il*_ = 1; *a*_*i*1_, …, *a*_*iK*_) as **π**_.**l**_, where *l* ∈ {00, 10, 01, 11} such that **π**_.00_ are the prior probabilities that the SNPs are null for both traits, **π**_.10_ are the prior probabilities that the SNPs are non-null for trait 1 and null for trait 2, **π**_.01_ are the prior probabilities that the SNPs are null for trait 1 and non-null for trait 2, and **π**_.11_ are the prior probabilities that the SNPs are non-null for both traits. Then assuming that the SNPs are independent, the joint distribution of the observed data *Pr*(**Y**, **A**) and the incomplete data log-likelihood (*ℓ*_*IC*_) and complete data log-likelihood (*ℓ*_*C*_) can be written as shown in Eqs 2, 3 and 4, respectively.

$$\begin{array}{c} \begin{array}{cc} Pr(Y,A) & =\prod_{i=1}^{M}[\sum_{l\in \{00,10,01,11\}}P\left(Z_{il}=1\right)P\left(Y_{i1},Y_{i2}|Z_{il}=1\right)]\\ & =\prod_{i=1}^{M}[\sum_{l\in \{00,10,01,11\}}\pi_{il}\;P\left(Y_{i1},Y_{i2}|Z_{il}=1\right)] \end{array} \end{array}$$ (2)

$$\begin{array}{c} \begin{array}{cc} \ell_{IC} & =\sum_{i=1}^{M}\;log[\sum_{l\in \{00,10,01,11\}}\pi_{il}\;P\left(Y_{i1},Y_{i2}|Z_{il}=1\right)] \end{array} \end{array}$$ (3)

$$\begin{array}{c} \begin{array}{cc} \ell_{C} & =\sum_{i=1}^{M}\sum_{l\in \{00,10,01,11\}}Z_{il}\;log[\pi_{il}\;P\left(Y_{i1},Y_{i2}|Z_{il}=1\right)] \end{array} \end{array}$$ (4)

### Algorithm

Given the approach described above, parameter estimation is implemented using an Expectation-Maximization (EM) algorithm [22]. The function *f* in Eq 1 is estimated by using a multivariate regression tree algorithm [21] that can identify combinations of functional annotations related to risk-associated SNPs for specific and multiple traits. The described approach is computationally implemented in two stages based on simulation study findings that showed improved parameter estimation and model stability when using a two-stage approach. Specifically, in Stage 1, we first estimate the parameters *α*_1_ and *α*_2_ without identifying a combination of functional annotations. Then, in Stage 2, we identify key combinations of functional annotations (*f*(**A**)) while the parameters *α*_1_ and *α*_2_ are kept fixed as the value obtained in Stage 1. Detailed calculation steps are illustrated below.

**Stage 1:** In Stage 1, we initialize $\alpha_{d}^{\left(0\right)}=0.1$, *d* = 1, 2 and $\pi_{il}^{\left(0\right)}=\frac{1}{2^{D}}$, *D* = 2 (the number of traits). In the *t*^*th*^ iteration of the E-step, define $Z_{il}^{\left(t\right)},\;l\in \left\{00,10,01,11\right\}$ for the *i*^*th*^ SNP as:

$$\begin{array}{c} \begin{array}{c} \;E-\text{step}:z_{il}^{\left(t\right)}=P\left(Z_{il}=1|Y,A;\;\theta^{\left(t-1\right)}\right) \end{array}\;=\frac{\pi_{il}^{\left(t-1\right)}\;P\left(Y_{i1},Y_{i2}|Z_{il}=1;\;\theta^{\left(t-1\right)}\right)}{\sum_{l^{\prime }\in \left\{00,10,01,11\right\}}\pi_{il^{\prime }}^{\left(t-1\right)}\;P\left(Y_{i1},Y_{i2}|Z_{il^{\prime }}=1;\;\theta^{\left(t-1\right)}\right)} \end{array}$$ (5)

In the *t*^*th*^ iteration of the M-step, **π**_*i*._, *α*_1_ and *α*_2_ are updated as:

$$\begin{array}{c} \begin{array}{c} M-\text{step}:\text{Fit}\;\text{a}\;\text{multivariate}\;\text{linear}\;\text{regression}\;\text{model}\;\text{as}\\ \;Z_{i.}^{\left(t\right)}=\beta_{0}^{\left(t\right)}+\beta_{1}^{\left(t\right)}a_{i1}+\cdots +\beta_{K}^{\left(t\right)}a_{iK}+{\epsilon_{i}}^{\left(t\right)}\\ \;\text{Update}\;\pi_{i.}\text{as}\;\text{the}\;\text{predicted}\;\text{value}\;\text{from}\;\text{the}\;\text{multivariate}\;\text{linear}\\ \;\text{regression}\;\text{model.}\\ \;\text{Update}\;\alpha_{1}^{\left(t\right)}=-\frac{\sum_{i=1}^{M}\left(z_{i10}^{\left(t\right)}\;+\;z_{i11}^{\left(t\right)}\right)}{\sum_{i=1}^{M}\left(z_{i10}^{\left(t\right)}\;+\;z_{i11}^{\left(t\right)}\right)\left(logY_{i1}\right)}\;\text{and}\;\alpha_{2}^{\left(t\right)}=-\frac{\sum_{i=1}^{M}\left(z_{i01}^{\left(t\right)}\;+\;z_{i11}^{\left(t\right)}\right)}{\sum_{i=1}^{M}\left(z_{i01}^{\left(t\right)}\;+\;z_{i11}^{\left(t\right)}\right)\left(logY_{i2}\right)} \end{array} \end{array}$$

where $\beta_{k}^{\left(t\right)},k=0,\cdots ,K$ are the regression coefficients and $\epsilon_{i}^{\left(t\right)}$ is the error term. The E and M steps are repeated until the incomplete log-likelihood and the *α*_1_ and *α*_2_ estimates converge. Then, *α*_1_, *α*_2_ and **π**_*i*._ estimated in this stage are used to fix *α*_1_, *α*_2_ and initialize **π**_*i*._, respectively, in Stage 2.

**Stage 2:** In stage 2, we implement another EM algorithm employing the multivariate regression tree algorithm, which allows for identification of union, intersection, and complement relationships between functional annotations in estimating **π**_*i*._. In the *t*^*th*^ iteration of the E-step, define $Z_{il}^{\left(t\right)},\;l\in \left\{00,10,01,11\right\}$ for the *i*^*th*^ SNP as shown in Eq 5, except *α*_1_ and *α*_2_ are fixed as $\hat{\alpha_{1}}$ and $\hat{\alpha_{2}}$, which are the final estimates of *α*_1_ and *α*_2_ obtained from Stage 1.

$$\begin{array}{c} \begin{array}{c} E-\text{step}:\text{Define}\;Z_{il}^{\left(t\right)},\;l\in \left\{00,10,01,11\right\}\;\text{as}\;\text{in}\;\text{Eq}\;5\text{,}\;\text{except}\;\alpha_{1}\;\text{and}\;\alpha_{2}\;\text{are}\;\\ \;\text{fixed}\;\text{as}\;\hat{\alpha_{1}}\;\text{and}\;\hat{\alpha_{2}}\text{,}\;\text{the}\;\text{final}\;\text{estimates}\;\text{of}\;\alpha_{1}\;\text{and}\;\alpha_{2}\;\text{from}\;\text{Stage}\;\text{1.} \end{array} \end{array}$$

In the *t*^*th*^ iteration of the M-step, **π**_*i*._ is updated as:

$$\begin{array}{c} \begin{array}{c} M-\text{step}:\text{Fit}\;\text{a}\;\text{multivariate}\;\text{regression}\;\text{tree}\;\text{model}\;\text{as}\;\text{shown}\;\text{below.}\\ \;Z_{i.}^{\left(t\right)}=f^{\left(t\right)}\left(a_{i1},\cdots ,a_{iK}\right)+\epsilon_{i}^{\left(t\right)},\;\text{where}\;\epsilon_{i}\;\text{is}\;\text{the}\;\text{error}\;\text{term}.\\ \;\text{Update}\;{\pi_{i.}}^{\left(t\right)}\;\text{as}\;\text{the}\;\text{predicted}\;\text{values}\;\text{from}\;\text{the}\;\text{multivariate}\;\text{regression}\\ \;\text{tree}\;\text{model.} \end{array} \end{array}$$ (6)

In the M-step, the complexity parameter (*cp*) of the multivariate regression tree is the key tuning parameter and defined as the minimum improvement that is required at each node of the tree. Specifically, in the multivariate regression tree model, the largest possible tree (i.e., a full-sized tree) is first constructed and then pruned using *cp*. This approach allows for the construction of the accurate yet interpretable multivariate regression tree that can explain relationships between functional annotations and risk-associated SNPs for one or more traits. The E and M steps are repeated until the incomplete log-likelihood converges. The pruned tree structure identified by the multivariate regression tree model upon convergence of the Stage 2 EM is the *f* in Eq 1.

We note that unlike the standard EM algorithm, the incomplete log-likelihood in Stage 2 is not guaranteed to be monotonically increasing. Therefore, we implement Stage 2 as a generalized EM algorithm by retaining only the iterations in which the incomplete log-likelihood increases compared to the previous iteration.

### Prioritization of marginal and joint risk-associated SNPs and identification of relevant functional annotations

Following parameter estimation, we can prioritize one or more trait risk associated SNPs using local false discovery rate or *fdr*. As shown in Eq 7, for marginal associations with a specific trait, we define *fdr* as the marginal posterior probability that the *i*^*th*^ SNP belongs to the non-risk-associated group for the specific trait given its GWAS association *p*-values for all traits and functional annotation information. Likewise, for joint associations between traits, we define *fdr* as the joint posterior probability that the *i*^*th*^ SNP belongs to the non-risk-associated group for the traits given its GWAS association *p*-values for all traits and functional annotation information. Next, we utilize the ‘direct posterior probability’ approach [23] to control the global false discovery rate (FDR).

$$\begin{array}{c} \begin{array}{c} fdr_{1}\left(Y_{i.},A_{i.}\right)=P\left(Z_{i00}+Z_{i01}=1|Y_{i.},A_{i.},\;\hat{\theta }\right)=\frac{P(Y_{i1},Y_{i2},Z_{i00}+Z_{i01}=1;\;\hat{\theta })}{P(Y_{i1},Y_{i2};\;\hat{\theta })},\\ fdr_{2}\left(Y_{i.},A_{i.}\right)=P\left(Z_{i00}+Z_{i10}=1|Y_{i.},A_{i.},\;\hat{\theta }\right)=\frac{P(Y_{i1},Y_{i2},Z_{i00}+Z_{i10}=1;\;\hat{\theta })}{P(Y_{i1},Y_{i2};\;\hat{\theta })},\\ fdr_{1,2}\left(Y_{i.},A_{i.}\right)=P\left(Z_{i00}+Z_{i10}+Z_{i01}=1|Y_{i.},A_{i.}\right)=\frac{P(Y_{i1},Y_{i2},Z_{i00}+Z_{i10}+Z_{i01}=1;\;\hat{\theta })}{P(Y_{i1},Y_{i2};\;\hat{\theta })}, \end{array} \end{array}$$

where

$$\begin{array}{c} \begin{array}{c} \text{where}\\ P\left(Y_{i1},Y_{i2};\;\hat{\theta }\right)=\sum_{l\in \{00,10,01,11\}}{\hat{\pi }}_{il}\;P\left(Y_{i1},Y_{i2}|Z_{il},{\text{A}}_{i.};\;\hat{\theta }\right), \end{array}\\ P(Y_{i1},Y_{i2},Z_{i00}+Z_{i01}=1;\;\hat{\theta })=\sum_{l\in \{00,01\}}{\hat{\pi }}_{il}\;P(Y_{i1},Y_{i2}|Z_{il},{\text{A}}_{i.};\;\hat{\theta }),\\ P(Y_{i1},Y_{i2},Z_{i00}+Z_{i10}=1;\;\hat{\theta })=\sum_{l\in \{00,10\}}{\hat{\pi }}_{il}\;P(Y_{i1},Y_{i2}|Z_{il},{\text{A}}_{i.};\;\hat{\theta }),\\ P(Y_{i1},Y_{i2},Z_{i00}+Z_{i10}+Z_{i01}=1;\;\hat{\theta })=\sum_{l\in \{00,10,01\}}{\hat{\pi }}_{il}\;P(Y_{i1},Y_{i2}|Z_{il},{\text{A}}_{i.};\;\hat{\theta }), \end{array}$$ (7)

Finally, relevant combinations of functional annotations are inferred based on the combination of functional annotations selected by the multivariate regression tree model upon convergence of the Stage 2 EM algorithm.

## Results

### Simulation study

**Fig 1:**
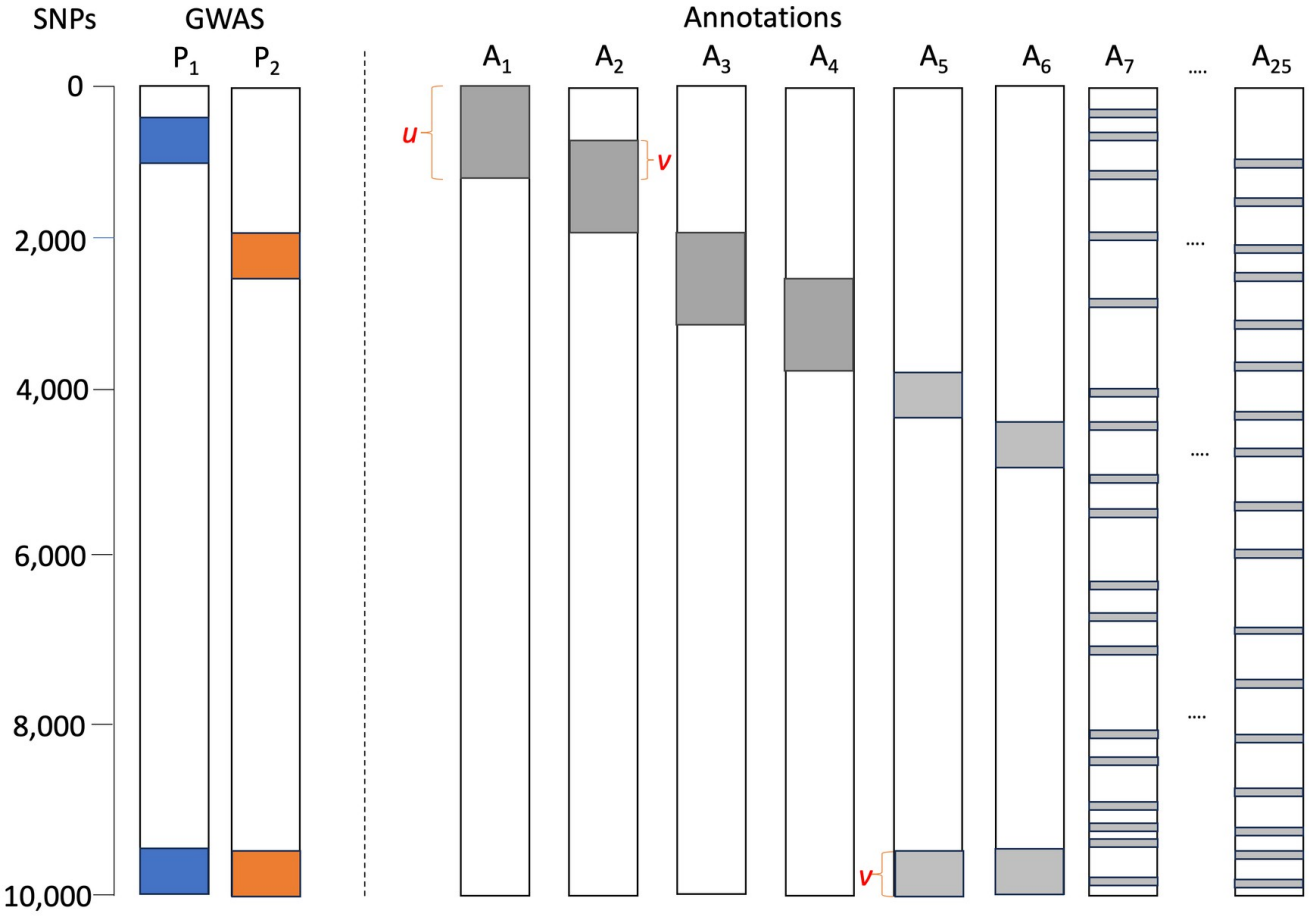
Simulation setting. The graphical scenario is presented for *M* = 10, 000 SNPs; *K* = 25 annotations; % of annotated SNPs in *A*_1_-*A*_6_ (*u*) = 10%; % of overlap between *A*_1_-*A*_2_, *A*_3_-*A*_4_, *A*_5_-*A*_6_ (*v*) = 50%; *A*_7_–*A*_15_ are noise SNPs, approximately 20% of which are randomly annotated; blue SNPs are non-null for trait *P*_1_ and their GWAS p-values are generated from *Beta*(*α*_1_ = 0.4, 1) distribution; orange SNPs are non-null for trait *P*_2_ and their GWAS p-values are generated from *Beta*(*α*_2_ = 0.4, 1) distribution; all other SNPs are null for both traits and their GWAS p-values are generated from *U*[0, 1] distribution for both traits.

We conducted a simulation study to evaluate the performance of the proposed multi-GPA-Tree approach. Fig 1 provides a graphical depiction of the simulation setting. For all simulation data, the number of SNPs was set to *M* = 10, 000, the number of annotations was set to *K* = 25, SNPs that are marginally associated with the first trait (*P*_1_) were assumed to be characterized with the combinations of functional annotations defined by *L*_1_ = *A*_1_ ∩ *A*_2_, SNPs that are marginally associated with the second trait (*P*_2_) were assumed to be characterized with the combinations of functional annotations defined by *L*_2_ = *A*_3_ ∩ *A*_4_, SNPs that are jointly associated with traits *P*_1_ and *P*_2_ were assumed to be characterized with the combinations of functional annotations defined by *L*_3_ = *A*_5_ ∩ *A*_6_, all the remaining functional annotations (*A*_*k*_, *k* = 7, …, 25) were considered to be noise annotations. Approximately 10% of SNPs were assumed to be annotated for annotations *A*_1_−*A*_6_, and *v*% where *v* = 35%, 50% and 75% of those annotated were assumed to overlap between the true combinations of functional annotations. For noise annotations *A*_7_−*A*_25_, approximately 20% of SNPs were annotated by first generating the proportion of annotated SNPs from *Unif*[0.1, 0.3] and then randomly setting this proportion of SNPs to one. For trait *P*_1_, the SNPs that satisfied the functional annotation combination in *L*_1_ or *L*_3_ were assumed to be risk-associated SNPs and their *p*-values were simulated from *Beta*(*α*_1_, 1) with *α*_1_ = 0.4. Similarly, for trait *P*_2_, the SNPs that satisfied the functional annotation combination in *L*_2_ or *L*_3_ were assumed to be risk-associated SNPs and their *p*-values were simulated from *Beta*(*α*_2_, 1) with *α*_2_ = 0.4. The SNPs that did not satisfy the required condition for association with *P*_1_ or *P*_2_ were assumed to be non-risk SNPs and their *p*-values were simulated from *U*[0, 1].

We simulated 50 datasets and compared the performance of multi-GPA-Tree with LPM [19] and GPA-Tree [20] using the simulation parameters defined above. Note that GPA-Tree can only integrate GWAS data for one trait with functional annotation data. Therefore, we fitted two separate GPA-Tree models for each of the two traits of interest and reported marginal association results for the two traits. The metrics for comparing the methods included (1) area under the curve (AUC) for marginal and joint associations, where the curve was created by plotting the true positive rate (sensitivity) against the false positive rate (1-specificity) to detect one or more trait risk-associated SNPs when global FDR was controlled at various levels; (2) statistical power to identify marginal and joint risk-associated SNPs when local FDR (*lfdr*) was controlled at the nominal level of 0.20; (3) predicted *lfdr* when *lfdr* was controlled at the nominal level of 0.20; and (4) estimation accuracy for *α*_*d*_ parameters in the *Beta*(*α*_*d*_, 1), *d* = 1, 2 distribution used to generate the *p*-values of risk-associated groups for traits *P*_1_ and *P*_2_. For multi-GPA-Tree, we also examined the accuracy of detecting the correct functional annotation tree based on (1) the proportion of simulation data for which all relevant functional annotations in *L*_1_, *L*_2_ and *L*_3_, i.e, annotation *A*_1_−*A*_6_, were identified simultaneously; (2) the average proportion of noise functional annotations (*A*_7_−*A*_25_) among the functional annotations identified by multi-GPA-Tree; and (3) the average proportion of true functional annotations (*A*_1_−*A*_6_) among the functional annotations identified by multi-GPA-Tree. Here we especially investigated how the overlap between SNPs annotated in *A*_1_−*A*_2_, *A*_3_−*A*_4_ and *A*_5_−*A*_6_ (*v*) impacted multi-GPA-Tree’s ability to separate relevant functional annotations from noise annotations for one or more trait risk-associated SNPs.

**Fig 2:**
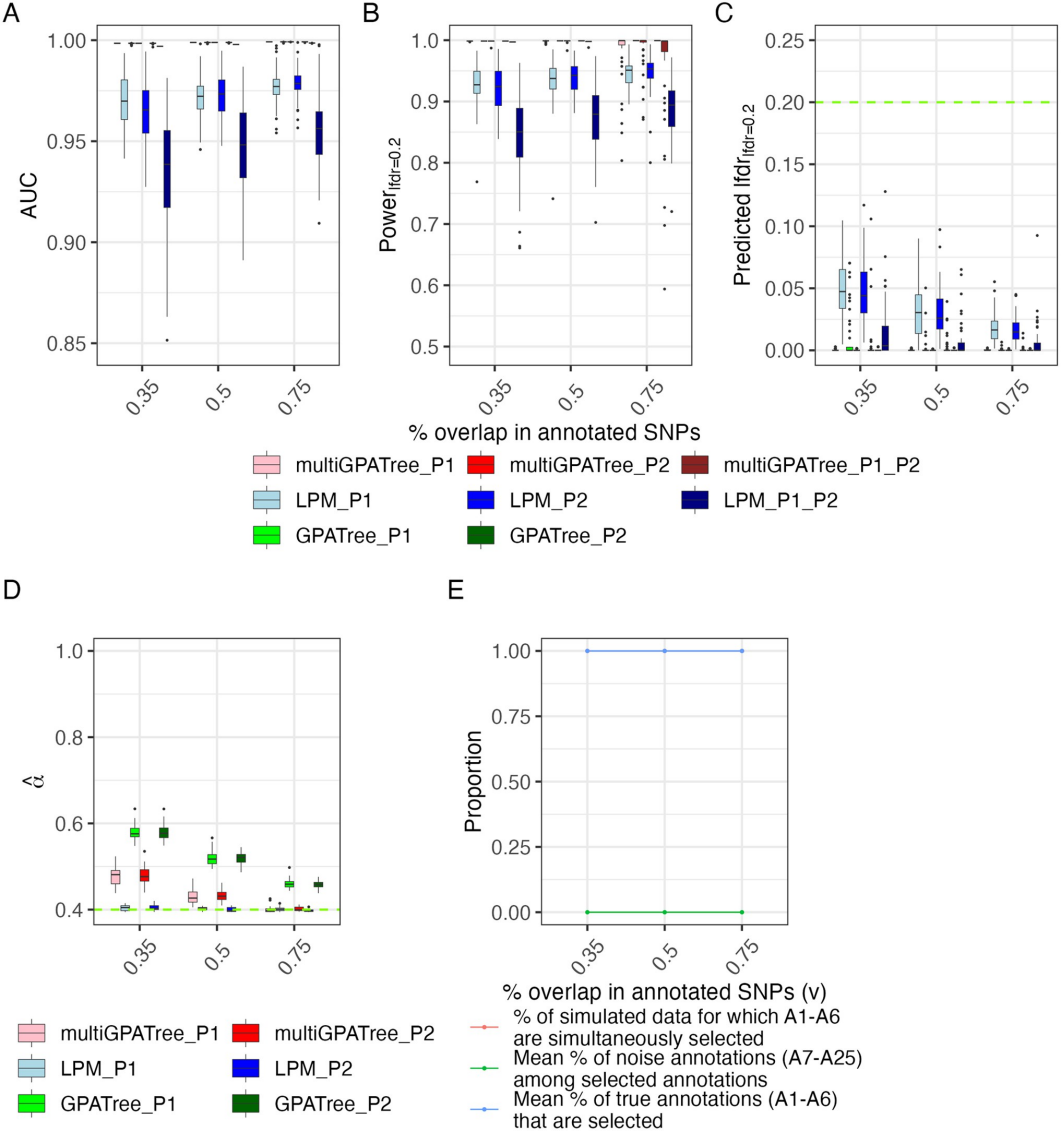
Simulation results. Comparison of (A) AUC, (B) statistical power to detect true marginal and joint risk-associated SNPs when local FDR (*lfdr*) is controlled at the nominal level of 0.20, (C) predicted local FDR (*lfdr*) when controlled at the nominal level of 0.20, (D) estimated *α*_1_ and *α*_2_ parameter for traits P1 and P2, and (E) proportion of simulated data for which only true annotations (*A*_1_−*A*_6_) are simultaneously selected (red line), the average proportion of noise annotations (*A*_7_−*A*_25_) among the functional annotations identified by multi-GPA-Tree (green line), and the average proportion of true annotations *A*_1_−*A*_6_ among the annotations identified by multi-GPA-Tree (blue line). We note that the blue and red lines are overlaid in the plot. The results are presented for different proportions of the overlap between SNPs annotated in *A*_1_−*A*_2_, *A*_3_−*A*_4_ and *A*_5_−*A*_6_ (*v*; x-axis). *M* = 10, 000, *K* = 25, and *α*_*d*_ = 0.4 in *Beta*(*α*_*d*_, 1), *d* = 1, 2. Results are summarized from 50 replications. Results related to marginal associations are presented using suffix *_P1 and *_P2 and results related to joint associations are presented using suffix *_P1_P2. Marginal and joint association results are presented for multi-GPA-Tree and LPM. Only marginal results are reported for GPA-Tree.

- **AUC:** Fig 2A compares the distribution of AUCs returned by multi-GPA-Tree, LPM and GPA-Tree. For all *v*, multi-GPA-Tree showed consistently higher AUC relative to LPM for both marginal and joint associations. LPM showed higher AUC for marginal associations relative to joint associations. For all *v*, GPA-Tree showed comparable AUC to multi-GPA-Tree for marginal associations.
- **Statistical power:** Fig 2B compares the distribution of power to detect true marginal and joint risk-associated SNPs when local FDR (*lfdr*) was controlled at 0.20 between multi-GPA-Tree, LPM and GPA-Tree. The multi-GPA-Tree approach showed higher statistical power to detect true marginal and joint risk-associated SNPs relative to LPM for all *v*. LPM showed higher power for marginal associations relative to joint association. LPM showed greater variability in statistical power compared to multi-GPA-Tree overall while multi-GPA-Tree showed more variability in power for higher *v*. For all *v*, GPA-Tree and multi-GPA-Tree showed comparable statistical power to detect true marginal associations.
- **Predicted local fdr (lfdr):** Fig 2C compares the distribution of predicted *lfdr* between multi-GPA-Tree, LPM and GPA-Tree when *lfdr* was controlled at the nominal level of 0.20. For all *v*, GPA-Tree and multi-GPA-Tree showed comparable low predicted *lfdr*. Although LPM showed higher predicted *lfdr* compared to multi-GPA-Tree and GPA-Tree, all three methods showed consistently controlled *lfdr* under 0.20 at the 0.20 level for all *v*.
- **Estimation of *α* parameters:** Fig 2D shows the distribution of *α* parameter estimates for traits 1 and 2 (P1 and P2) using multi-GPA-Tree, LPM and GPA-Tree. LPM was on average more accurate than multi-GPA-Tree and GPA-Tree, and multi-GPA-Tree was more accurate than GPA-Tree in estimating *α* for both traits. Both multi-GPA-Tree and GPA-Tree approach generally overestimated *α* and this was most notable for smaller *v*. As *v* increased, *α* estimates from multi-GPA-Tree became closer to the true value. We note that overestimation of *α* by multi-GPA-Tree did not impact the method’s ability to identify the true combinations of functional annotations or the marginal and joint risk-associated SNPs, which are the main objectives of multi-GPA-Tree.
- **Selection of relevant and noise annotations:** The red line in Fig 2E shows the proportion of times only functional annotations in the true combination *L*_1_, *L*_2_ and *L*_3_ (*A*_1_ − *A*_6_) were simultaneously identified by multi-GPA-Tree. The red line aligned exactly with the blue line which shows the mean proportion of true annotations (*A*_1_−*A*_6_) among all selected annotations. Finally, the green line shows the proportion of noise annotations (*A*_7_ − *A*_25_) among the selected annotation. The alignment of the red and blue lines and the horizontal green line at 0 suggest that only and all relevant annotations were selected by multi-GPA-Tree. These results demonstrate the potential of multi-GPA-Tree to correctly identify true annotations from noise annotations.

Additional simulation results assuming shared functional annotation between marginally and jointly associated SNPs are presented in Section A in S1 Text. Two distinct simulation scenarios are assumed: 1) jointly associated SNPs share the same functional annotation as marginally associated SNPs while also depending on other unique annotations, and 2) jointly associated SNPs share the same functional annotation as marginally associated SNP without depending on any other unique functional annotations. Overall, multi-GPA-Tree showed better performance compared to both LPM and GPA-Tree while also correctly identifying relevant annotations from noise annotations under both scenarios.

### Real data application

**Fig 3:**
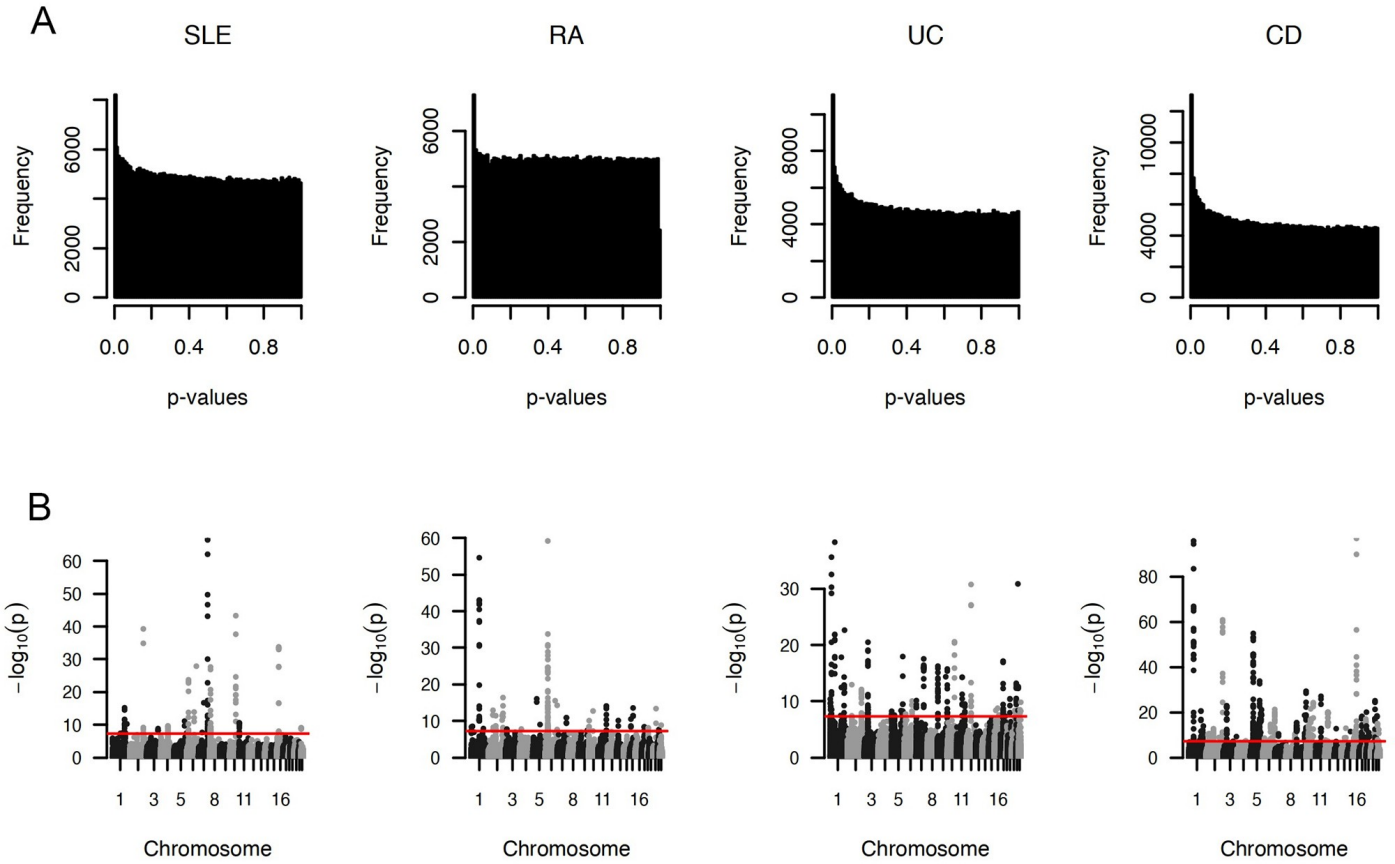
GWAS summary statistic plots. (A) GWAS p-value histogram and (B) Manhattan plots for the four GWAS. Genome-wide significance level (−*log*_10_(5 × 10^−8^)) is indicated by the red line.

We obtained a combined dataset including the SLE [24] and RA [25], and CD and UC [26] GWAS. Summary statistics in the SLE and RA GWAS was profiled for 18, 264 (6, 748 cases and 11, 516 controls) and 58, 284 (14, 361 cases and 43, 923 controls) individuals of European ancestry, respectively. Summary statistics in the CD and UC GWAS was profiled from 8, 467 (4, 686 cases and 3, 781 controls) individuals of European ancestry. Following quality control and exclusion of SNPs in the MHC region, approximately 492, 557 SNPs were utilized in the final analysis and integrated with functional annotation data from GenoSkyline (GS) [27] and GenoSkylinePlus (GSP) [28]. The Manhattan plots and *p*-value histogram plots for the four GWAS data are presented in Fig 3A and 3B, respectively.

**Fig 4:**
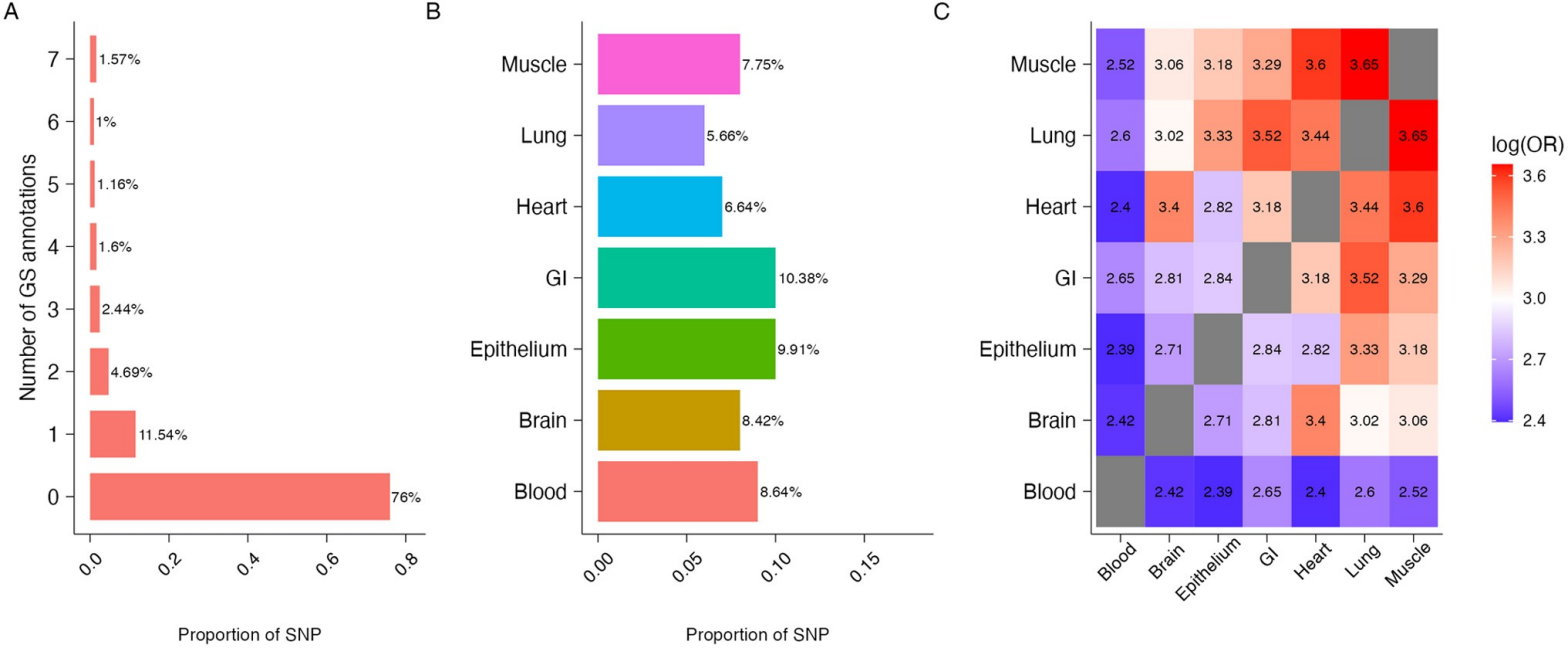
Characteristics of 492, 557 SNPs when integrated with seven GenoSkyline (GS) annotations. (A) Number of GS tissues in which SNPs are annotated. (B) Proportion of SNPs that are annotated for each GS tissue type. (C) Overlap of SNPs annotated by seven GS tissue types, calculated using log odds ratio.

**Fig 5:**
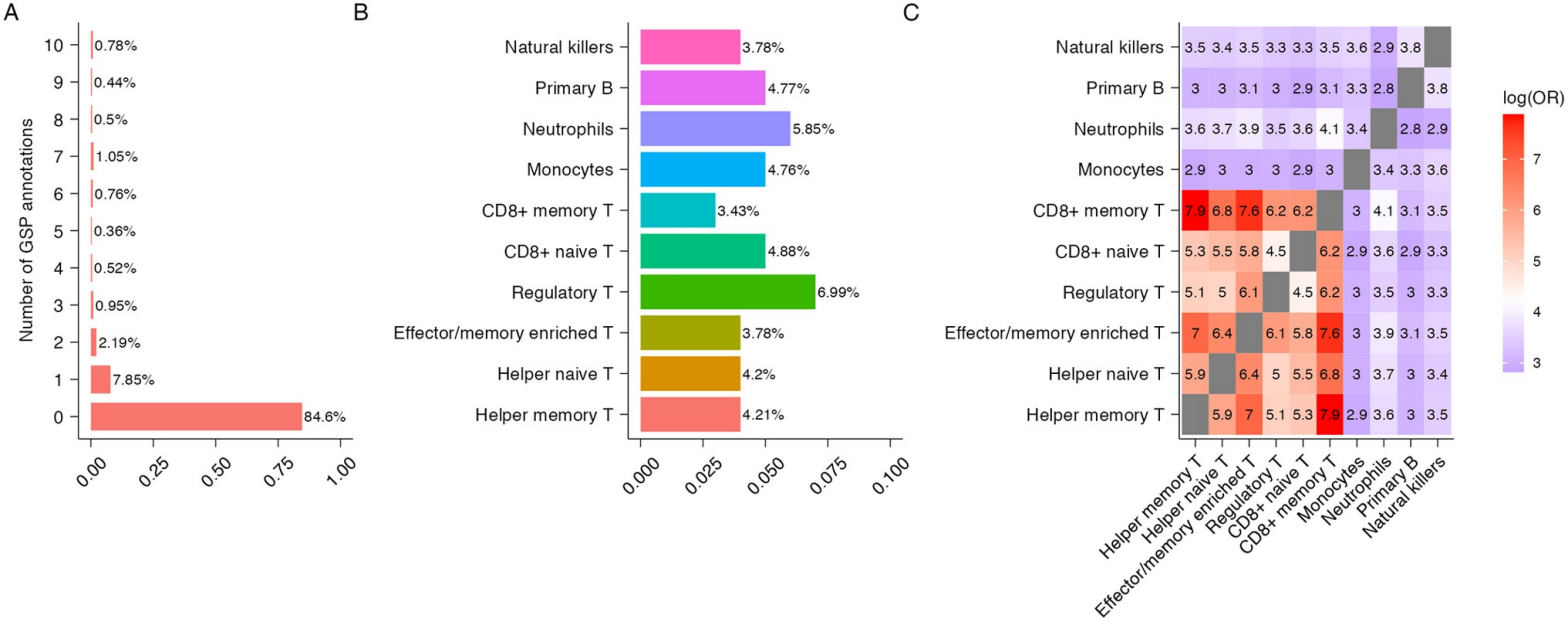
Characteristics of 492, 557 SNPs when integrated with 10 blood related GenoSkylinePlus (GSP) annotations. (A) Number of GSP tissues in which SNPs are annotated. (B) Proportion of SNPs that are annotated for each blood related GSP annotations. (C) Overlap of SNPs annotated by 10 blood related GPS annotations, calculated using log odds ratio.

We descriptively investigated the functional potential of the 492, 557 SNPs using seven tissue-specific GS annotations (Fig 4) and ten blood-related cell-type specific GSP annotations (Fig 5). With a GS and GSP score cutoff of 0.5, 24% of SNPs were annotated in at least one of the seven tissue types (Fig 4A) and 15.4% of SNPs were annotated in at least one of the 10 blood related cell-type specific annotations (Fig 5A). The percentage of annotated SNPs ranged from 5.66% for lung tissue to 10.38% for GI tissue (Fig 4B) and from 3.43% for primary T CD8^+^ memory cells to 6.99% for primary T regulatory cells (Fig 5B). We also measured the overlap in SNPs annotated in different tissue-types and cell-types using log odds ratio (Figs 4C and 5C). Consistent with the literature stipulating that muscle and lung tissues show higher levels of eQTL sharing while blood shows the lowest [27, 29], our findings show that SNPs annotated for muscle, lung and heart tissues overlap more with other tissue types while SNPs annotated for blood tissue overlap less (Fig 4C). Finally, we observed the different types of T cells (Primary helper memory, helper naive, effector/memory enriched, regulatory, CD8^+^ naive and CD8^+^ memory T cells) overlap more with each other while neutrophils, primary B and natural killer cells overlap less (Fig 5C).

### Integration of systemic lupus erythematosus (SLE) and rheumatoid arthritis (RA) GWAS

#### Tissue-level investigation using GenoSkyline (GS) annotations

We applied the multi-GPA-Tree approach to the SLE and RA GWAS and tissue-specific GS annotations to identify SNPs that are marginally and jointly associated with SLE and RA, and to characterize the functional annotations relevant to single and multiple trait risk-associated SNPs. At the nominal global FDR level of 0.05, multi-GPA-Tree identified 394 SNPs that are jointly associated with both SLE and RA, 1087 SNPs that are marginally associated with SLE and 791 SNPs that are marginally associated with RA (Table 1).

**Table 1:** Real data application results summary. Number of jointly and marginally associated SNPs when systemic lupus erythematosus (SLE) and rheumatoid arthritis (RA), and Crohn’s disease (CD) and ulcertive colitis (UC) GWAS are integrated with the GenoSkyline (GS) and GenoSkylinePlus (GSP) annotations when jointly and individually analyzed using the multi-GPA-Tree, the LPM and the GPA-Tree approach. All analysis included 492, 557 SNPs and 7 tissue-specific GS and 10 blood-related cell-type specific GSP annotations. Inference for marginal and joint associations are based on global FDR control at the nominal level of 0.05. For LPM, annotation enrichment is done separately for P1 and P2, and reported annotations are those with p-value <0.05 for both P1 and P2.

Data integration	Approach	# marginally associated with P1 (SLE/UC)	# marginally associated with P2 (RA/CD)	# jointly associated with P1 and P2 (SLE+RA/ UC+CD)	Selected annotation	Computation time
SLE+RA+GS	multi-GPA-Tree	1, 087	791	394	Blood	5.97 mins
SLE+RA+GS	LPM	1, 120	794	412	Blood GI	34.1 mins
SLE+GS	GPA-Tree	696	-	-	Blood	9.44 mins
RA+GS	GPA-Tree	-	470	-	Blood	12.8 mins
SLE+RA+GSP	multi-GPA-Tree	1, 065	760	383	Regulatory T	9.38 mins
SLE+RA+GSP	LPM	1, 101	769	410	Primary B Regulatory T Helper memory T CD8+ memory T	35.8 mins
SLE+GSP	GPA-Tree	830	-	-	Primary B Regulatory T Helper memory T	8.43 mins
RA+GSP	GPA-Tree	-	634	-	Regulatory T Helper memory T Natural killer	11.3 mins
UC+CD+GS	multi-GPA-Tree	5, 430	5, 041	5, 041	Blood	15.9 mins
UC+CD+GS	LPM	5, 183	4, 926	4, 613	Blood Brain Epithelium GI	36.1 mins
UC+GS	GPA-Tree	1, 566	-	-	Blood	8.08 mins
CD+GS	GPA-Tree	-	3, 185	-	Blood	3.52 mins
UC+CD+GSP	multi-GPA-Tree	4, 995	4, 912	4, 576	Natural killer	17.1 mins
UC+CD+GSP	LPM	5, 116	4, 878	4, 567	Primary B Regulatory T Helper memory T Effector memory enriched T CD8+ naive T CD8+ memory T Monocytes Neutrophils Natural Killers	36.2 mins
UC+GSP	GPA-Tree	1, 654	-	-	Natural killer Monocytes Effector/Memory enriched T Regulatory T	6.59 mins
CD+GSP	GPA-Tree	-	3, 232	-	Natural killer Monocytes Effector/Memory enriched T Primary B Regulatory T	5.87 mins

**Fig 6:**
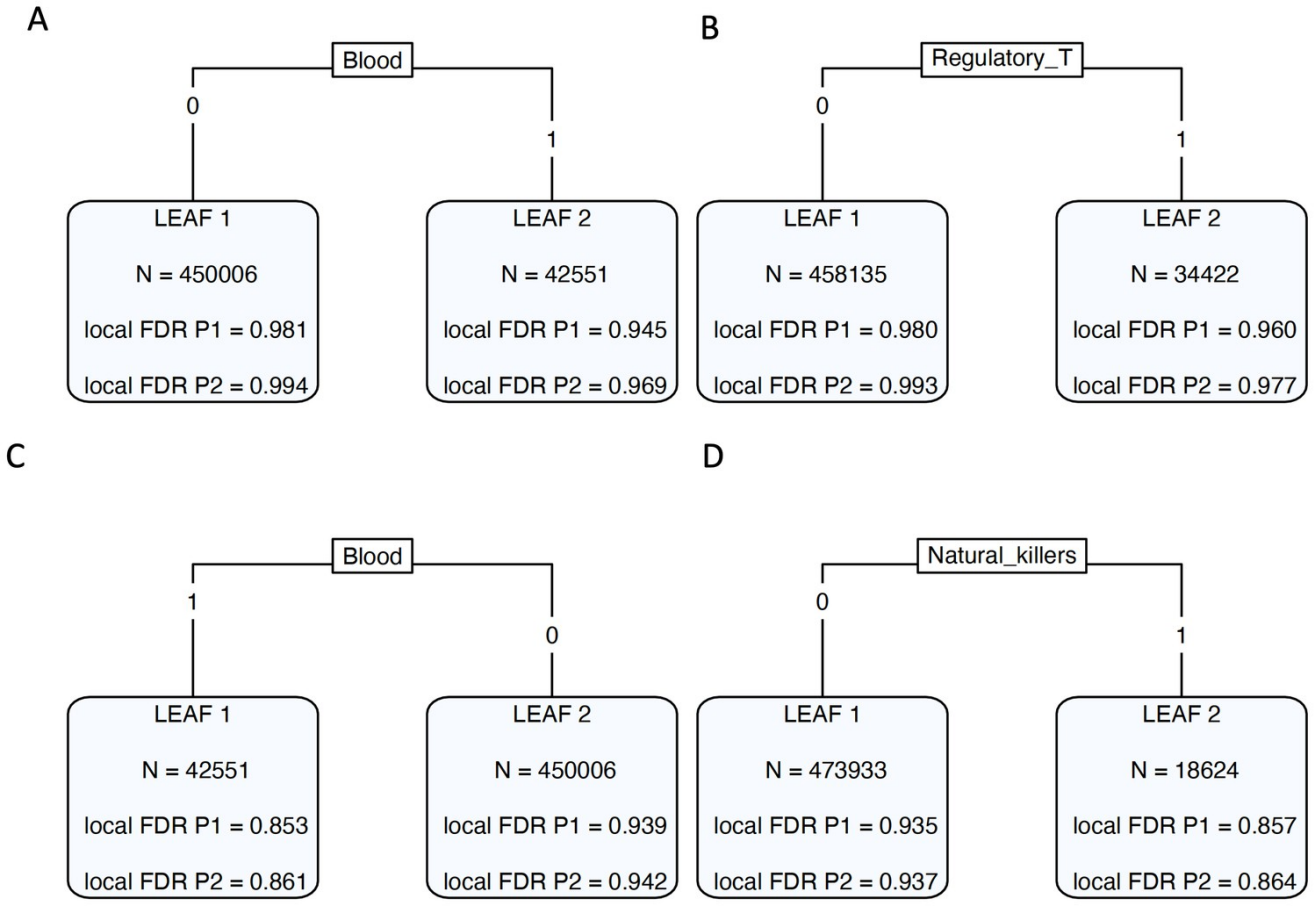
Real data application results. Trees returned by multi-GPA-Tree models when (A) SLE (P1), RA (P2) and GenoSkyline annotations are integrated, (B) SLE (P1), RA (P2) and GenoSkylinePlus annotations are integrated, (C) UC (P1), CD (P2) and GenoSkyline annotations are integrated, and (D) UC (P1), CD (P2) and GenoSkylinePlus annotations are integrated.

In the joint analysis of SLE and RA with tissue-specific GS annotations, the original multi-GPA-Tree model identified blood tissue at the root node and included 2 leaves (Fig 6A). Further investigation showed that 156 SNPs that were jointly associated with both SLE and RA, 336 SNPs that were marginally associated with SLE and 306 SNPs that were marginally associated with RA were also annotated for blood tissue. Of the 156 jointly associated SNPs that were also annotated for blood tissue, 118 SNPs were protein-coding such that chromosomes 1, 6, 2 and 17 had the most number of protein-coding SNPs. The *PLCL1* gene in chromosome 2, *IL2RA* gene in chromosome 10 and *UHRF1BP1* gene in chromosome 6 had the most number of protein-coding SNPs with 5 SNPs related to coding the *PLCL1* gene and 4 SNPs each related to coding the *IL2RA* and *UHRF1BP1* genes. The *PLCL1* gene is known to promote inflammatory response by regulating the NLRP3 inflammasomes, a component of the immune system related to activation and secretion of proinflammatory cytokines [30]. Similarly, *IL2RA* gene expression has been reported on activated T and B cells, regulatory T cells, activated monocytes, and natural killer cells [31, 32], and the *UHRF1BP1* gene plays a role in non-conservative amino-acid change and is related to RNA processing complex that is targeted by SLE autoantibodies [33].

We also discovered 3 SNPs each in chromosomes 4, 17, 3, 3, and 16 known to code the *BANK1*, *PGAP3*, *PLCL2*, *RASA2*, and *TXNDC11* genes, respectively. *BANK1* is primarily expressed in CD19^+^ B cells and is a known SLE and RA susceptibility gene [34, 35]; in animal studies, the *PGAP3* gene knockout has been associated with reduced apoptotic cell clearance, a causal pathway for autoimmunity [36]; *PLCL2* is known to encode a negative regulator of B cell receptor signalling important in controlling immune responses and is a known susceptibility gene for RA [37]. Finally, although not explored in the context of SLE and RA, *RASA2* variants are known to be associated with combined allergy diseases [38] and *TXNDC11* is known to play a role in thyroid hormone biosynthesis [39].

We additionally implemented the GPA-Tree approach by integrating the GS annotations to the SLE and RA GWAS individually, and also implemented the LPM approach to jointly analyze SLE and RA GWAS. Validating our multi-GPA-Tree results, blood tissue was identified at the root node in the separate GPA-Tree analysis for both SLE and RA, and blood tissue along with GI tissue was also identified by LPM for both SLE and RA. In the individual GPA-Tree analysis, we identified 696 SNPs to be associated with SLE and 470 SNPs to be associated with RA with 229 SLE associated and 224 RA associated SNPs also annotated for blood tissue. 655 of those associated with SLE, and 450 of those associated with RA were also found to be marginally associated with SLE and RA, respectively, using multi-GPA-Tree (Table A in S1 Text). Of the top 3 genes identified in the joint analysis of SLE and RA, one or more protein-coding SNPs related to the *IL2RA* and *PLCL1* genes were also identified in the single trait analysis of both SLE and RA by GPA-Tree. However, protein-coding SNPs related *UHRF1BP1* gene were identified for SLE but not for RA in single trait analysis by GPA-Tree. Likewise, LPM identified 1, 120 SNPs to be marginally associated with SLE, 794 SNPs to be marginally associated with RA and 412 SNPs to be jointly associated with SLE and RA, replicating 1, 076 SLE associated marginal SNPs, 780 RA associated marginal SNPs, and 388 SLE and RA associated joint SNPs also identified by multi-GPA-Tree.

#### Cell-type-level investigation using GenoSkylinePlus (GSP) annotations

Based on the observed relationship between GS annotation for blood tissue and SLE and RA, in the second phase of the analysis, we applied the multi-GPA-Tree approach to the SLE and RA GWAS and 10 blood related cell-type specific GSP annotations to identify SNPs that were marginally and jointly associated with SLE and RA, and to characterize the blood related GSP functional annotations relevant to single and multiple trait risk-associated SNPs. At the nominal global FDR level of 0.05, multi-GPA-Tree identified 383 SNPs that were jointly associated with SLE and RA, 1, 065 SNPs that were marginally associated with SLE and 760 SNPs that were marginally associated with RA (Table 1). The joint analysis also identified primary T regulatory cells at the root node (Fig 6B) with 95 SNPs that were jointly associated with both SLE and RA, 191 SNPs that were marginally associated with SLE and 176 SNPs that were marginally associated with RA also annotated for regulatory T cells. Of the 95 jointly associated SNPs that were also annotated for regulatory T cells, 69 were protein coding such that chromosomes 1, 6, 16 and 2 had the most number of protein-coding SNPs. The *PLCL1* gene in chromosome 2, *IL2RA* gene in chromosome 10 and *TXNDC11* gene in chromosome 16 had the most number of protein-coding SNPs with 3 different protein-coding SNPs related to coding each of the 3 genes.

The individual analysis using the GPA-Tree approach identified primary B, regulatory T and helper memory T cells with primary B cell at the root node for SLE. Similarly, we identified regulatory T, helper memory T and natural killer cells with regulatory T cells at the root node for RA. Similarly, LPM also identified primary B, regulatory T, helper memory T and CD8+ memory T cells for both SLE and RA. In the individual GPA-Tree analysis, we identified 830 SNPs to be associated with SLE and 634 SNPs to be associated with RA. 650 of those associated with SLE, and 437 of those associated with RA were also found to be marginally associated with SLE and RA, respectively, using multi-GPA-Tree (Table A in S1 Text). Of those associated with SLE, 176 were annotated for primary B, 122 were annotated for regulatory T, and 43 were annotated for helper memory T cells. Among SNPs associated with RA, 132 were annotated for both regulatory T and natural killer cells, 148 were annotated for regulatory T and not for natural killer cells, 32 were annotated for natural killer but not for regulatory T cells and 35 were annotated for helper memory T cells. Among the top 3 genes identified in the joint analysis of SLE, RA and GSP annotations, one or more protein-coding SNPs related to the *IL2RA* gene were also identified in the single trait analysis of both SLE and RA. However, protein-coding SNPs related to the *PLCL1* gene were identified for SLE only while protein-coding SNPs related to the *TXNDC11* gene were identified for RA only. Likewise, LPM identified 1, 101 SNPs to be marginally associated with SLE, 769 SNPs to be marginally associated with RA and 410 SNPs to be jointly associated with SLE and RA, replicating 1, 043 SLE associated marginal SNPs, 742 RA associated marginal SNPs, and 372 SLE and RA associated joint SNPs also identified by multi-GPA-Tree.

### Integration of ulcerative colitis (UC) and Crohn’s disease (CD) GWAS

#### Tissue-level investigation using GenoSkyline (GS) annotations

We also applied the multi-GPA-Tree approach to the UC and CD GWAS and tissue-specific GS annotations to identify SNPs that were marginally and jointly associated with UC and CD, and to characterize the functional relevance of the single and multiple trait risk-associated SNPs. At the nominal global FDR level of 0.05, multi-GPA-Tree identified 5, 041 SNPs that were jointly associated with both UC and CD, 5, 430 SNPs that were marginally associated with UC and 5, 041 SNPs that were marginally associated with CD (Table 1). In this joint analysis, the original multi-GPA-Tree model identified blood tissue at the root node and included 2 leaves (Fig 6C). Further investigation showed that 1, 319 SNPs that were jointly associated with both UC and CD, 1, 453 SNPs that were marginally associated with UC and 1, 319 SNPs that were marginally associated with CD were also annotated for blood tissue. Of the 1, 319 jointly associated and blood annotated SNPs, 990 were protein-coding. Chromosomes 1 and 2 had the most number of protein-coding SNPs, followed by chromosomes 17 and 5. The *THADA* and *ATG16L1* genes in chromosome 2, *C5orf56* gene in chromosome 5 and *IKZF3* gene in chromosome 17 had the most number of protein-coding SNPs with 9 SNPs each related to coding the *THADA* and *IKZF3* genes, and 8 SNPs each related to coding the *ATG16L1* and *C5orf56* genes. Although not directly implicated in the pathogenesis of UC or CD, the *THADA* gene is known to influence metabolic mechanisms like adipogenesis [40]. In contrast, genetic variants of the *ATG16L1* gene are some of the most studied in the pathogenesis of Crohn’s disease, playing a role in pathogen clearance, cytokine production, protein regulation and endoplasmic stress response control [41, 42]. Similarly, increased expression of *IKZF3*, a transcription factor that plays an important role in the regulation of B lymphocyte proliferation and differentiation, has been observed in patients with CD and UC [43, 44], and *C5orf56* is known to influence the immune stimulus specific enhancer for *IRF1*, a gene established in the pathogenesis of Crohn’s disease [45–47].

We also discovered 7 SNPs each in chromosomes 5, 6 and 9 known to code the *FYB*, *BACH2* and *DOCK8* genes, and 6 SNPs each known to code the *BANK1*, *LEF1*, and *NFKB1* genes in chromosome 4. The *FYB* gene is related to T cells signaling and plays a role in IL-2A expression, and is known to be associated with some autoimmune regulation [48, 49]. Likewise, *BACH2* is a critical gene for B cell regulatory activity and T cell function and differentiation and is a known susceptibility locus for CD and UC [50, 51]; *DOCK8* is known to regulate diverse immune sub-types including lymphocytes and plays a role in immune synapse formation and pathogen proliferation [52]; and *NFKB1* is a known transcription regulator of immune response, apoptosis and cell proliferation and is up-regulated in both UC and CD patients [53]. On the contrary, although *BANK1* is a B cell gene known to be associated with SLE and RA [34, 35], only few studies linking specific *BANK1* variants to CD has been published [54, 55] and it’s role in the pathogenesis of both CD and UC remains understudied. This is also true for the *LEF1* gene, a known mediator in the Wnt signaling pathway [56].

In the individual trait analysis for UC and CD using the GPA-Tree approach, we identified blood, GI and epithelium tissues for UC, and blood and epithelium tissues for CD with blood tissue at the root node for both traits. Blood, GI, epithelium and brain tissues were also identified by LPM for both UC and CD. GPA-Tree identified 1, 566 SNPs to be associated with UC and 3, 185 SNPs to be associated with CD with 540 UC associated and 960 CD associated SNPs also annotated for blood tissue. Although multi-GPA-Tree identified a significantly large number of marginally associated variants for both UC and CD relative to GPA-Tree, GPA-Tree replicated a large number of marginal associations also identified by multi-GPA-Tree such that 1, 386 of those found to be associated with UC and 2, 787 of those found to be associated with CD using GPA-Tree were also identified by multi-GPATree (Table A in S1 Text). Among the top 4 genes identified in the joint analysis of UC, CD and GS annotations, one or more SNPs related to the *THADA*, *IKZF3* and *C5orf56* genes were also identified in the single trait analysis of both UC and CD. However, SNPs related to *ATG16L1* gene were identified for CD only. Likewise, LPM identified 5, 183 SNPs to be marginally associated with UC, 4, 926 SNPs to be marginally associated with CD and 4, 613 SNPs to be jointly associated with UC and CD, replicating 5, 136 UC associated marginal SNPs, 4, 868 CD associated marginal SNPs, and 4, 603 UC and CD associated joint SNPs also identified by multi-GPA-Tree.

#### Cell-type-level investigation using GenoSkylinePlus (GSP) annotations

In the second phase of the analysis, we combined the UC and CD GWAS and 10 blood related cell-type specific GSP annotations using the multi-GPA-Tree approach. At the nominal global FDR level of 0.05, multi-GPA-Tree identified 4, 576 SNPs that were jointly associated with UC and CD, 4, 995 SNPs that were marginally associated with UC and 4, 912 SNPs that were marginally associated with CD (Table 1). The original multi-GPA-Tree model fit identified primary natural killer cells at the root node and included 2 leaves (Fig 6D). Further investigation showed that 507 SNPs that were jointly associated with both UC and CD, 579 SNPs that were marginally associated with UC and 554 SNPs that were marginally associated with CD were annotated for natural killer cells. Of the 507 jointly associated and natural killer cells annotated SNPs, 360 were protein-coding. Chromosomes 1 and 2 had the most number of protein-coding SNPs, followed by chromosomes 5 and 17. The *C5orf56* and *IRF1* genes in chromosome 5 and *FAM53B* gene in chromosome 10 had the most number of protein-coding SNPs with 8 SNPs related to coding the *C5orf56* gene, 5 SNPs related to coding the *IRF1* gene and 4 SNPs related to coding the *FAM53B* gene. We also discovered 3 SNPs each known to code the *ATG16L1* and *THADA* genes in chromosome 2, *IKZF3* and *PGAP3* genes in chromosome 17, *DOCK8* gene in chromosome 9, *TSPAN14* gene in chromosome 10 and *ETS1* gene in chromosome 11. *FAM53B* is known to be associated with humoral immune reponse, regulation of immune effector process, and regulation of lymphocyte activation [57]; reduced expression of *PGAP3* is known to be related to impaired clearance of apoptotic cells and has been observed in CD and UC patients [44]; *TSPAN14* is expressed in immune cell types participating in immunity and inflammation, and is positively correlated with microphages and neutrophils and negatively correlated with T cells CD8 [58]; and finally, *ETS1* is known to be over-expressed in intestinal epithelial cells of patients with UC [59], and has also been linked to fistula formation, an epithelial defect caused by destructive inflammation, in the pathogenesis of CD [60].

The individual analysis using the GPA-Tree approach identified primary natural killer, monocytes, effector/memory enriched T and regulatory T cells with natural killer cells at the root node for both UC and CD. Additionally, primary B cells was also identified for CD. In addition to identifying primary B, regulatory T, natural killer, monocytes and effector/memory enriched T for both UC and CD, LPM also identified helper memory T, CD8+ naive T, CD8+ memory T and neutrophil to be associated with both UC and CD. In the individual GPA-Tree analysis, we identified 1, 654 SNPs to be associated with UC and 3, 232 SNPs to be associated with CD. GPA-Tree replicated many of the multi-GPA-Tree findings such that 1, 361 of those associated with UC and 2, 755 of those associated with CD using GPA-Tree were also found to be marginally associated with UC and CD, respectively, using multi-GPA-Tree (Table A in S1 Text). Of those associated with UC using GPA-Tree, 186 were annotated for both natural killer and effector/memory enriched T cells, 134 were annotated for natural killer cells but not for effector/memory enriched T cells, 112 were annotated for monocytes and 127 were annotated for regulatory T cells. Similarly, of those associated with CD using GPA-Tree, 278 were annotated for both natural killer and effector/memory enriched T cells, 211 were annotated for natural killer cells but not for effector/memory enriched T cells, 181 were annotated for monocytes, 161 were annotated for regulatory T and 132 were annotated for primary B cells. Among the top 3 genes identified in the joint analysis of UC, CD and GSP annotations, one or more protein-coding SNPs related to the *C5orf56*, *IRF1* and *FAM53B* genes were also identified in the single trait analysis of both UC and CD. Likewise, LPM identified 5, 116 SNPs to be marginally associated with UC, 4, 878 SNPs to be marginally associated with CD and 4, 567 SNPs to be jointly associated with UC and CD, replicating 4, 933 UC associated marginal SNPs, 4, 758 CD associated marginal SNPs, and 4, 438 UC and CD associated joint SNPs also identified by multi-GPA-Tree.

## Discussion

Over the past 20 years, several GWAS have been conducted, leading to successful identification of over two hundred thousand trait risk-associated genetic variants [1]. The advancement in complexity of newer statistical approaches to exploit the richness in GWAS data even further has been helpful in identifying many previously unknown genetic variants and it is expected that newer discoveries are forthcoming. Current findings have been crucial in identifying treatment therapies and for new drug discoveries [61–63]. Yet, a crucial gap that needs to be filled with new variant discovery is in our understanding of the functional mechanisms and pathways through which genetic variants influence traits. It is well known that complex traits are often caused by an amalgamation of functional mechanisms that can be described by multiple functional annotations [64, 65]. Therefore, identifying the combinations of functional annotations that are associated with the traits can provide valuable insight into trait etiology. However, to the best of our knowledge, we are currently lacking statistical methodologies that identify the combinations of functional annotations that act in unison to influence traits. We propose the discussed multi-GPA-Tree approach to fill in this gap.

In comparison to existing methods, the overall strength of the multi-GPA-Tree approach is that it can automatically select the combinations of functional annotations from a group of annotations without excessively increasing the complexity of the model and be used to benefit our understanding of the functional mechanisms related to a single or multiple traits. The multi-GPA-Tree approach achieves that goal by following a hierarchical architecture that combines an iterative procedure (EM algorithm) and a multivariate decision tree algorithm. During simulation study, the multi-GPA-Tree approach showed consistently better performance than the LPM approach in terms of AUC, statistical power and type-I error control in identifying trait risk-associated variants for single and multiple traits and also distinctly identified relevant annotations from noise annotations with great accuracy (Fig 2). Moreover, multi-GPA-Tree also showed higher computational efficiency in real data application such that it was consistently faster to implement than LPM under all data integration scenarios. We note that although GPA-Tree seems faster to implement than multi-GPA-Tree at first glance, a more accurate representation of “total computational time” for GPA-Tree is to add the computational time taken to analyze the two traits separately. As such, multi-GPA-Tree was faster to implement than GPA-Tree for SLE and RA, while GPA-Tree was faster to implement than multi-GPA-Tree for UC and CD.

We compared the real data application findings from multi-GPA-Tree to findings from LPM and also our recently published method ‘GPA-Tree’ [20], a statistical approach that does not exploit the pleiotropic relationship between traits and prioritizes variants that are marginally associated with a single trait. Our comparison demonstrated that the performance of multi-GPA-Tree was similar to that of LPM with respect to the number of marginally and jointly associated SNPs identified by the two methods such that LPM replicated majority of the SNPs also identified by multi-GPA-Tree. However, multi-GPA-Tree was more conservative in identifying the combinations of annotations, largely identifying the subset of annotations identified by LPM. On the contrary, compared to GPA-Tree, multi-GPA-Tree consistently identified more marginally risk-associated variants for both traits. This difference was more prominent in the joint analysis of UC, CD and GenoSkylinePlus annotations using GPA-Tree and multi-GPA-Tree (Table A in S1 Text). Further evaluation of these results showed that, out of the 3, 634 unique variants identified to be marginally associated with UC using multi-GPA-Tree, 2, 021 were protein coding such that 28 variants are known to code for the *MUC19* gene in chromosome 12, 19 variants are known to code for the *THADA* gene in chromosome 2, 18 variants are known to code for the *CDKAL1* gene in chromosome 6, and 15 variants are known to code for the *AGBL4* gene in chromosome 1. Similarly, out of the 2, 157 unique variants identified to be marginally associated with CD using multi-GPA-Tree, 1, 181 were protein coding such that 14 variants are known to code for the *ABGL4* gene in chromosome 1, 13 variants are known to code for the *USP34* and *CADM2* genes in chromosomes 2 and 3, respectively, and 10 variants are known to code for the *BANK1* gene in chromosome 4. These results suggests that multi-GPA-Tree might potentially identify additional functional variants related to trait etiology. Evidently, while GPA-Tree identified more annotations to be relevant with a specific trait, multi-GPA-Tree identified annotations that are largely common between the two jointly analyzed traits. For instance, blood tissue was identified in both joint and individual analysis of SLE and RA, and UC and CD. Similarly, regulatory T cells was identified as a relevant annotation when SLE and RA were jointly analyzed which was also a common annotation identified when SLE and RA are individually analyzed. Likewise, natural killer cells was identified as a relevant annotation when UC and CD were jointly analyzed, again a common annotation also identified for both UC and CD when individually analyzed. Overall, these results are consistent with previous literature indicating connections between autoimmune diseases like SLE, RA, UC and CD and blood tissue [66–68], and SLE and RA and regulatory T cells [69–73], and UC and CD and natural killer cells [74–77]. Moreover, in addition to identifying a few candidate genes (*RASA2, TXNDC11, THADA*) for SLE, RA, UC and CD that have previously been linked to other allergy, thyroid or metabolic traits, we also validated previous findings linking the *PLCL1*, *IL2RA* and *UHRF1BP1* genes to SLE and RA [33, 78–84], and the *ATG16L1, C5orf56* and *IKZF3* genes to UC and CD [44–46, 85–89].

From the statistical modeling perspective, several assumptions are made in multi-GPA-Tree. First, we assume that the genetic variants are conditionally independent given its functional information which greatly simplifies our model and leads to efficient computation of the parameter estimates. Although this assumption omits the linkage disequilibrium (LD) structure present between SNPs in the same genomic region, it still allows us to conservatively infer risk-associated variants by modestly controlling the type-I error rate by potentially also identifying SNPs that are in LD with each other to be risk-associated. Second, we assume that signal in the GWAS association p-values are related to the functional potential of a SNP, so some functional signal should be present in the GWAS and annotation data for the multi-GPA-Tree approach to work efficiently. Simulation results suggest that at least 10% of variants should be functionally annotated for at least one feature to achieve valid parameter estimates and controlled type-I error at the nominal level.

Our approach has some limitations. First, in our two-stage estimation approach for multi-GPA-Tree, we made two modifications from the standard EM algorithm in Stage 2: (1) we implemented a generalized EM algorithm, which increases the incomplete likelihood in each iteration; and (2) we implemented a “constrained optimization” that fixes *α* at the value obtained in Stage 1 throughout iterations. This approach may have two shortcomings: 1) the final estimate of *α* may not be the optimal global solution, and 2) uncertainty of *α* might not be fully represented in Stage 2. As such, theoretical convergence cannot be guaranteed. Yet, we observed that this approach provides more robust estimation results in our simulation studies in terms of AUC, statistical power, false discovery rate control, and accuracy of the identified decision tree. Investigation of the theoretical properties for our model framework is an area of future investigation. Second, the current implementation of multi-GPA-Tree is designed so that only pleiotropic relationship between two GWAS traits can be exploited at a time due to computational challenges. We plan to investigate expansion to exploit pleiotropic relationship between more than two GWAS traits in the future.

Here we have presented a novel statistical approach, named multi-GPA-Tree, that can exploit pleiotropic relationship between multiple GWAS data and integrate GWAS data and tissue and cell-type specific functional annotation data in an efficient manner. Compared to some existing approaches which require genotype data at the individual level and annotation data that follows certain distributional assumption, multi-GPA-Tree only requires summary statistics for GWAS data and binary annotation data for analysis. These features make multi-GPA-Tree an attractive and effective tool for the integrative analysis of GWAS data with functional annotation data. Despite the promising statistical improvements made by multi-GPA-Tree, the biological implications need to be independently replicated and newly identified variants need to be independently validated. Two limitations of multi-GPA-Tree are that it cannot handle continuous or count annotation data and scaling multi-GPA-Tree to more than two traits can still be computationally challenging. Addressing issues related to integrating multiple GWAS and multiple types of annotation data are important areas of our future work.

## Supporting information

S1 Text Supporting information for multi-GPA-Tree. Additional simulation study and real data application results.
(PDF)
