# Supplementary material for: multi-GPA-Tree: Statistical approach for pleiotropy informed and functional annotation tree guided prioritization of GWAS results
Source: PLoS Comput Biol. 2023 Dec 7;19(12):e1011686. doi: 10.1371/journal.pcbi.1011686 (PMC10729974; doi:10.1371/journal.pcbi.1011686)
Supplement: S1 Text — Additional simulation study and real data application results. (PDF) [file pcbi.1011686.s001.pdf]

S1 Text: Supporting Information for "multi-GPA-Tree: Statistical Approach for Pleiotropy Informed and Functional Annotation Tree Guided Prioritization of GWAS Results"

Aastha Khatiwada[1], Ayse Selen Yilmaz[2], Bethany J. Wolf[3], Maciej Pietrzak[2], Dongjun Chung[2,4,*]*

[1]Divison of Biostatistics and Bioinformatics, National Jewish Health, Denver, Colorado, USA
[2]Department of Biomedical Informatics, The Ohio State University, Columbus, Ohio, USA
[3]Department of Public Health Sciences, Medical University of South Carolina, Charleston, South Carolina, USA
[4]Pelotonia Institute for Immuno-Oncology, The James Comprehensive Cancer Center, The Ohio State University, Columbus, Ohio, USA

[*]To whom correspondence should be addressed (chung.911@osu.edu).

A Additional Simulation Studies to Investigate Performance of multi-GPA-Tree

1 Assuming jointly associated SNPs share the same functional annotation as marginally associated SNPs while also depending on some unique annotations

In this section, we evaluated the performance of the proposed multi-GPA-Tree approach assuming jointly associated SNPs share the same functional annotation as marginally associated SNPs while also depending on some unique functional annotations. Fig **??** provides a graphical depiction of the simulation setting. For all simulation data, the number of SNPs was set to $M = 10,000$, the number of annotations was set to $K = 25$, SNPs that are marginally associated with the first trait (P_1) were assumed to be characterized with the combinations of functional annotations defined by $L_1 = A_1 \cap A_2$, SNPs that are marginally associated with the second trait (P_2) were assumed to be characterized with the combinations of functional annotations defined by $L_2 = A_3 \cap A_4$, SNPs that are jointly associated with traits P_1 and P_2 were assumed to be characterized with the combinations of functional annotations defined by $L_3 = (A_1 \cap A_5) \cup (A_5 \cap A_6)$, all the remaining functional annotations ($A_k, k = 7, \ldots, 25$) were considered to be noise annotations. Approximately 10% of SNPs were assumed to be annotated for annotations $A_1 - A_6$, and % of overlap between A_1 and A_2, A_1 and A_5, and A_5 and A_6 is $\frac{1}{2}v$ and % of overlap between A_3 and A_4 is v where $v = 35\%, 50\%$ and 75% of those annotated. For noise annotations A_7-A_{25}, approximately 20% of SNPs were annotated by first generating the proportion of annotated SNPs from $Unif[0.1, 0.3]$ and then randomly setting this proportion of SNPs to one. For trait P_1, the SNPs that satisfied the functional annotation combination in L_1 or L_3 were assumed to be risk-associated SNPs and their p-values were simulated from $Beta(\alpha_1, 1)$ with $\alpha_1 = 0.4$. Similarly, for trait P_2, the SNPs that satisfied the functional annotation combination in L_2 or L_3 were assumed to be risk-associated SNPs and their p-values were simulated from $Beta(\alpha_2, 1)$ with $\alpha_2 = 0.4$. The SNPs that did not satisfy the required condition for association with P_1 or P_2 were assumed to be non-risk SNPs and their p-values were simulated from $U[0, 1]$.

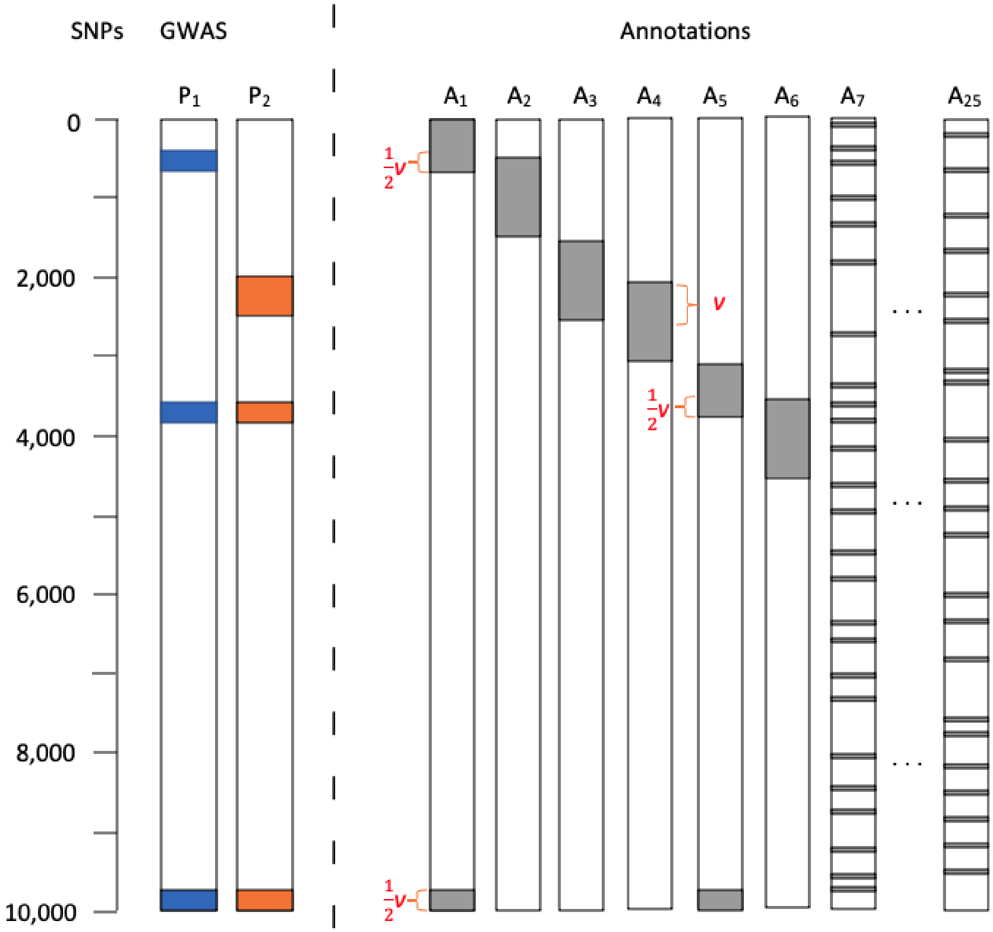

Figure A: **Simulation setting.** The graphical scenario is presented for $M = 10,000$ SNPs; $K = 25$ annotations; % of annotated SNPs in A_1-A_6 $(u) = 10\%$; % of overlap between A_1 and A_2, A_1 and A_5, and A_5 and A_6 is $\frac{1}{2}v$ and % of overlap between A_3 and A_4 is v, where $v = 50\%$; A_7–A_{15} are noise SNPs, approximately 20% of which are randomly annotated; blue SNPs are non-null for trait P_1 and their GWAS p-values are generated from $Beta(\alpha_1 = 0.4, 1)$ distribution; orange SNPs are non-null for trait P_2 and their GWAS p-values are generated from $Beta(\alpha_2 = 0.4, 1)$ distribution; all other SNPs are null for both traits and their GWAS p-values are generated from $U[0, 1]$ distribution for both traits.

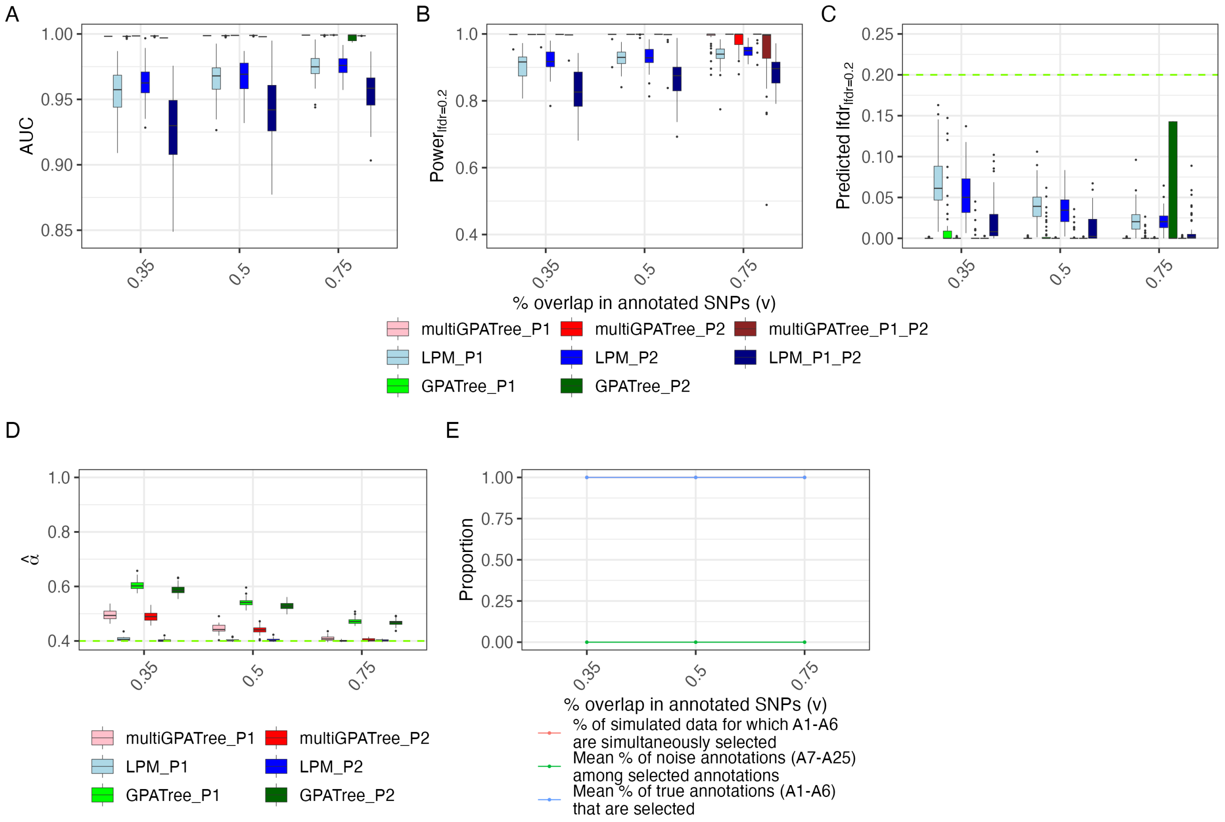

Figure B: **Simulation results.** Comparison of (A) AUC, (B) statistical power to detect true marginal and joint risk-associated SNPs when local FDR ($lfdr$) is controlled at the nominal level of 0.20, (C) predicted local FDR ($lfdr$) when controlled at the nominal level of 0.20, (D) estimated α_1 and α_2 parameter for traits P1 and P2; and (E) proportion of simulated data for which only true annotations ($A_1 - A_6$) are simultaneously selected (red line), the average proportion of noise annotations ($A_7 - A_{25}$) among the functional annotations identified by multi-GPA-Tree (green line), and the average proportion of true annotations $A_1 - A_6$ among the annotations identified by multi-GPA-Tree (blue line). We note that the blue and red lines are overlaid in the plot. The results are presented for different proportions of the overlap between SNPs annotated in $A_1 - A_2$, $A_3 - A_4$ and $A_5 - A_6$ (v; x-axis). $M = 10,000$, $K = 25$, and $\alpha_d = 0.4$ in $Beta(\alpha_d, 1), d = 1, 2$. Results are summarized from 50 replications. Results are summarized from 50 replications. Marginal association results are presented using suffix *_P1 and *_P2 and joint association results are presented using suffix *_P1_P2. Only marginal results are reported for GPA-Tree.

2 Assuming jointly associated SNPs share the same functional annotation as marginally associated SNPs without depending on any other unique functional annotations

Next, we evaluated the performance of the proposed multi-GPA-Tree approach assuming jointly associated SNPs share the same functional annotation as marginally associated SNPs without depending on any other unique functional annotations. Fig **??** provides a graphical depiction of the simulation setting. For all simulation data, the number of SNPs was set to $M = 10,000$, the number of annotations was set to $K = 25$, SNPs that are marginally associated with the first trait (P_1) were assumed to be characterized with the combinations of functional annotations defined by $L_1 = A_1 \cap A_2$, SNPs that are marginally associated with the second trait (P_2) were assumed to be characterized with the combinations of functional annotations defined by $L_2 = A_3 \cap A_4$, SNPs that are jointly associated with traits P_1 and P_2 were assumed to be characterized with the combinations of functional annotations defined by $L_3 = A_1 \cap A_5$, all the remaining functional annotations ($A_k, k = 6, \ldots, 25$) were considered to be noise annotations. Approximately 10% of SNPs were assumed to be annotated for annotations $A_1 - A_5$ while A_1 has an additional v% annotated SNPs. The % of overlap between A_1 and A_2, A_3 and A_4, and A_1 and A_5 is v where $v = 35\%, 50\%$ and 75% of those annotated. For noise annotations $A_6 - A_{25}$, approximately 20% of SNPs were annotated by first generating the proportion of annotated SNPs from $Unif[0.1, 0.3]$ and then randomly setting this proportion of SNPs to one. For trait P_1, the SNPs that satisfied the functional annotation combination in L_1 or L_3 were assumed to be risk-associated SNPs and their p-values were simulated from $Beta(\alpha_1,\ 1)$ with $\alpha_1 = 0.4$. Similarly, for trait P_2, the SNPs that satisfied the functional annotation combination in L_2 or L_3 were assumed to be risk-associated SNPs and their p-values were simulated from $Beta(\alpha_2,\ 1)$ with $\alpha_2 = 0.4$. The SNPs that did not satisfy the required condition for association with P_1 or P_2 were assumed to be non-risk SNPs and their p-values were simulated from $U[0,\ 1]$.

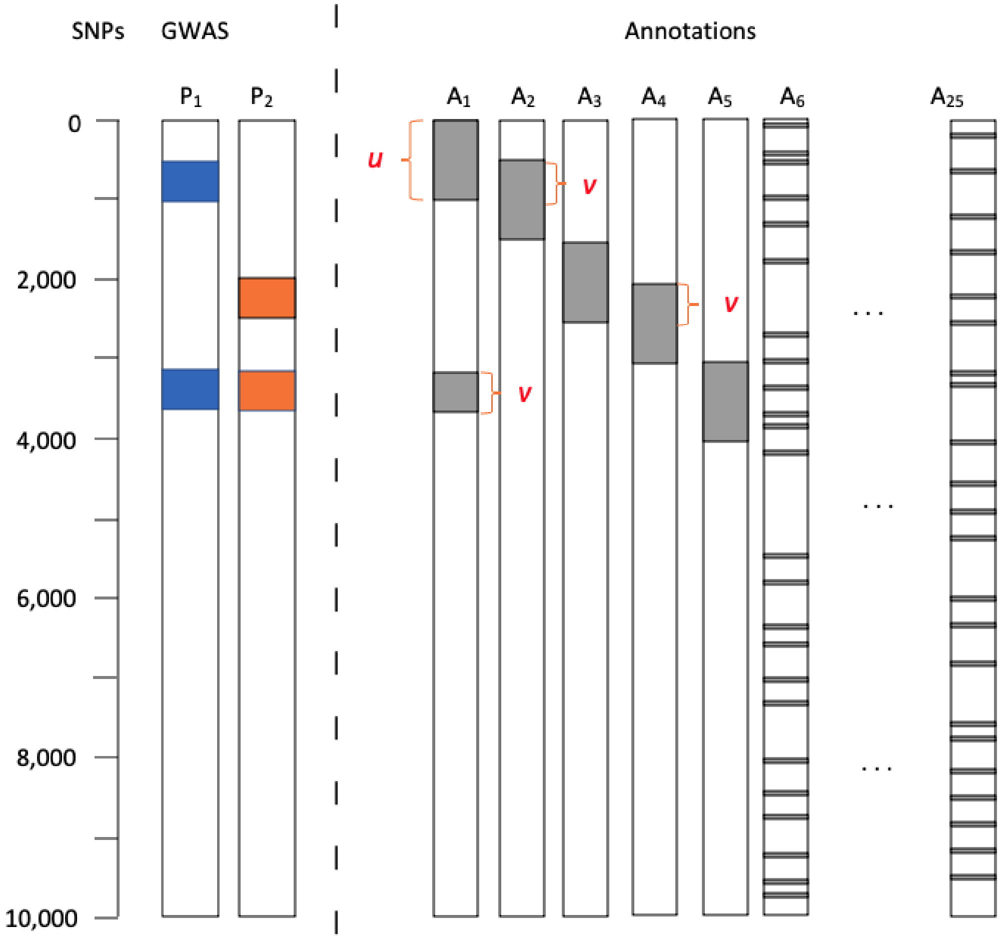

Figure C: **Simulation setting.** The graphical scenario is presented for $M = 10,000$ SNPs; $K = 25$ annotations; 10% of SNPs are annotated for annotations $A_1 - A_5$ while A_1 has an additional v% annotated SNPs; % of overlap between A_1 and A_2, A_3 and A_4, and A_1 and A_5 is v, where $v = 50\%$; A_6–A_{25} are noise SNPs, approximately 20% of which are randomly annotated; blue SNPs are non-null for trait P_1 and their GWAS p-values are generated from $Beta(\alpha_1 = 0.4, 1)$ distribution; orange SNPs are non-null for trait P_2 and their GWAS p-values are generated from $Beta(\alpha_2 = 0.4, 1)$ distribution; all other SNPs are null for both traits and their GWAS p-values are generated from $U[0, 1]$ distribution for both traits.

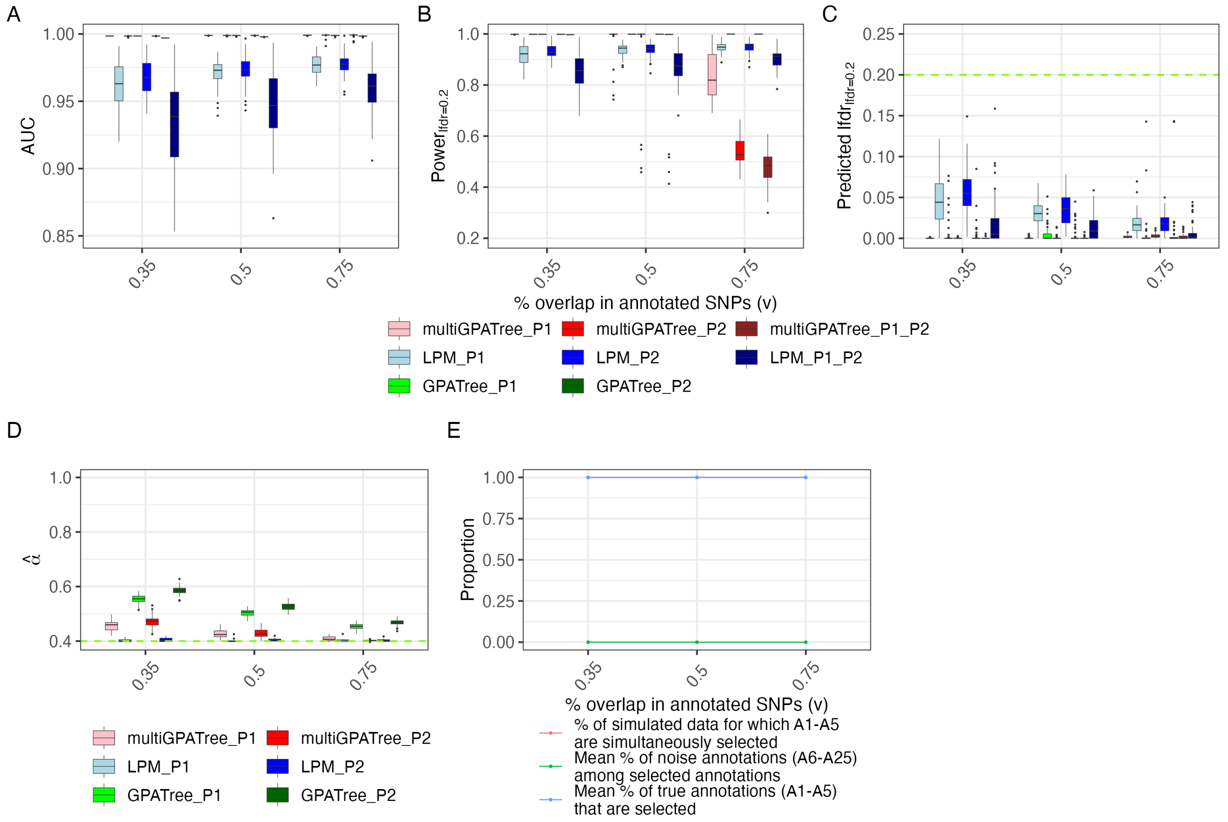

Figure D: **Simulation results.** Comparison of (A) AUC, (B) statistical power to detect true marginal and joint risk-associated SNPs when local FDR ($lfdr$) is controlled at the nominal level of 0.20, (C) predicted local FDR ($lfdr$) when controlled at the nominal level of 0.20, (D) estimated α_1 and α_2 parameter for traits P1 and P2; and (E) proportion of simulated data for which only true annotations ($A_1 - A_5$) are simultaneously selected (red line), the average proportion of noise annotations ($A_6 - A_{25}$) among the functional annotations identified by multi-GPA-Tree (green line), and the average proportion of true annotations $A_1 - A_5$ among the annotations identified by multi-GPA-Tree (blue line). We note that the blue and red lines are overlaid in the plot. The results are presented for different proportions of the overlap between SNPs annotated in A_1 and A_2, A_3 and A_4, and A_1 and A_5 (v; x-axis). $M = 10,000$, $K = 25$, and $\alpha_d = 0.4$ in $Beta(\alpha_d, 1), d = 1, 2$. Results are summarized from 50 replications. Marginal association results are presented using suffix *_P1 and *_P2 and joint association results are presented using suffix *_P1_P2. Only marginal results are reported for GPA-Tree.

B Additional Real Data Application Results

1 Frequency of risk-associated SNPs that are common and unique to multi-GPA-Tree and GPA-Tree

	Integration with GS			Integration with GSP		
	SLE	RA	SLE+RA	SLE	RA	SLE+RA
1) multi-GPA-Tree, GPA-Tree	655	450	-	650	437	-
2) multi-GPA-Tree	432	341	394	415	323	383
3) GPA-Tree	41	20	-	180	197	-
	UC	CD	UC+CD	UC	CD	UC+CD
1) multi-GPA-Tree, GPA-Tree	1,386	2,787	-	1,361	2,755	-
2) multi-GPA-Tree	4,044	2,254	5,041	3,634	2,157	4,576
3) GPA-Tree	180	398	-	293	477	-

Table A: **Common and unique risk-associated SNPs.** Number of marginally and jointly associated SNPs that are 1) common between multi-GPA-Tree and LPM, 2) unique to multi-GPA-Tree, and 3) unique to GPA-Tree.
